# Structural and biochemical characterisation of the N‐carbamoyl‐β‐alanine amidohydrolase from *Rhizobium radiobacter* MDC 8606

**DOI:** 10.1111/febs.16943

**Published:** 2023-09-08

**Authors:** Ani Paloyan, Armen Sargsyan, Mariam D. Karapetyan, Artur Hambardzumyan, Sergei Kocharov, Henry Panosyan, Karine Dyukova, Marina Kinosyan, Anna Krueger, Cecilia Piergentili, Will A. Stanley, Karrera Y. Djoko, Arnaud Baslé, Jon Marles‐Wright, Garabed Antranikian

**Affiliations:** ^1^ Scientific and Production Center “Armbiotechnology” of NAS RA Yerevan Armenia; ^2^ The Scientific Technological Centre of Organic and Pharmaceutical Chemistry SNPO of NAS RA Yerevan Armenia; ^3^ Authority for the Environment, Climate, Energy and Agriculture in Hamburg Hamburg Germany; ^4^ School of Natural and Environmental Sciences Newcastle University Newcastle upon Tyne UK; ^5^ Department of Biosciences Durham University Durham UK; ^6^ Newcastle University Biosciences Institute, Faculty of Medical Sciences Newcastle University Newcastle upon Tyne UK; ^7^ Center for Biobased Solutions TUHH Hamburg Germany

**Keywords:** carbamoylase, metalloprotein, N‐carbamoyl‐β‐alanine, X‐ray crystallography, β‐ureidopropionase

## Abstract

N‐carbamoyl‐β‐alanine amidohydrolase (CβAA) constitutes one of the most important groups of industrially relevant enzymes used in the production of optically pure amino acids and derivatives. In this study, a CβAA‐encoding gene from *Rhizobium radiobacter* strain MDC 8606 was cloned and overexpressed in *Escherichia coli*. The purified recombinant enzyme (RrCβAA) showed a specific activity of 14 U·mg^−1^ using N‐carbamoyl‐β‐alanine as a substrate with an optimum activity at 55 °C and pH 8.0. In this work, we report also the first prokaryotic CβAA structure at a resolution of 2.0 Å. A discontinuous catalytic domain and a dimerisation domain attached through a flexible hinge region at the domain interface have been revealed. We identify key ligand binding residues, including a conserved glutamic acid (Glu131), histidine (H385) and arginine (Arg291). Our results allowed us to explain the preference of the enzyme for linear carbamoyl substrates, as large and branched carbamoyl substrates cannot fit in the active site of the enzyme. This work envisages the use of RrCβAA from *R. radiobacter* MDC 8606 for the industrial production of L‐α‐, L‐β‐ and L‐γ‐amino acids. The structural analysis provides new insights on enzyme–substrate interaction, which shed light on engineering of CβAAs for high catalytic activity and broad substrate specificity.

## Graphical abstract

*Rhizobium radiobacter* N‐carbamoyl‐β‐alanine amidohydrolase (RrCβAA) catalyses the metal‐dependent decarbamoylation of N‐carbamoyl‐amino acids. RrCβAA was recombinantly produced in *Escherichia coli* and purifies with bound Zn^2+^; however, the protein is most active with Mn^2+^ as a cofactor. The RrCβAA enzyme is highly active at 55˚C and is stable up to 65 °C. It can catalyse the decarbamoylation of a range of linear N‐carbamoyl amino acids and has potential uses to produce high‐value enantiomerically pure amino acids.

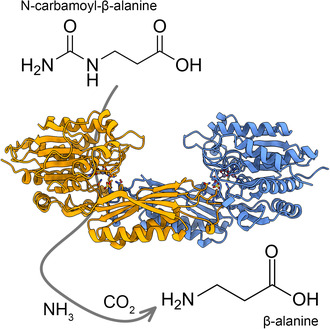

**Abbreviations**
BCA: bicinchoninic acid
DTNB: 5,5′‐dithiobis‐(2‐nitrobenzoic acid)
DTT: dithiothreitol
EDTA: ethylenediaminetetraacetic acid
ICP‐MS: inductively coupled plasma mass spectrometry
LB: Lysogeny Broth
MDC: Microbial Depository Center
MES: 2‐(N‐morpholino)ethanesulfonic acid
MMT: DL‐malic acid, MES, Tris
OPA: o‐phthalaldehyde
PDB: Protein Data Bank
RrCβAA: *Rhizobium radiobacter* MDC8606 N‐carbamoyl‐β‐alanine amidohydrolase
TCA: trichloroacetic acid
Tris: tris(hydroxymethyl)aminomethane

## Introduction

Optically pure L‐amino acids find many industrial uses, where they are used as feed and food additives and as intermediates for pharmaceuticals, cosmetics, and pesticides [1]. While there are only 20 standard proteinogenic amino acids, hundreds of amino acids have been identified in nature or have been chemically synthesised [2]. Although they are less abundant than their proteinogenic L‐α‐analogues, natural and synthetic L‐β‐, L‐γ‐ and L‐δ‐ amino acids have found applications in the pharmaceutical industry, such as diaminobutyric acid [3], as well as in different fields of biotechnology, such as those being used to investigate the structure and dynamics of proteins, to study protein interactions and to modulate the activity of proteins in living cells [4]. β‐Amino acids have been used as building blocks of peptides, peptidomimetics and many other physiologically active compounds [5]. For example, β‐alanine is used as a dietary supplement, especially by athletes, for its potential activity in the formation of the dipeptides anserine and carnosine [6], which may improve cerebral blood flow and verbal episodic memory [7]. Another example is the well‐known γ‐aminobutyric acid and its derivatives, which are widely used as health supplements [8]. δ‐Amino acids are particularly valuable as chemical precursors; for example, 5‐aminovalerate is a C5 platform chemical used in the synthesis of δ‐valerolactam [9], glutarate [10], and as a precursor for nylon fibres [11], and resins [12].

In the last decade, chemical synthesis of these amino acids has received considerable research attention, and several reviews on catalytic asymmetric synthesis strategies can be found [13]. From the biotechnological point of view, among the amino acid production technologies, the hydantoinase process is distinguished as a multienzyme and ecologically friendly process, which guarantees absolute stereospecificity in the production of amino acids [14]. With this method, the potential production of any optically pure amino acids from a wide spectrum of D‐, L‐5‐monosubstituted hydantoins has proven to be viable [1, 15]. The method is widely used to produce L‐ and D‐ amino acids by using L‐N‐ (E.C. 3.5.1.87) or D‐N‐carbamoylase enzymes (E.C. 3.5.1.77), which convert N‐carbamoyl‐amino acids to their corresponding optically pure amino acids in the last stage of the hydantoinase process [16, 17]. Characterisation of prokaryotic N‐carbamoyl‐β‐alanine amidohydrolase enzymes (NCβAAs, E.C. 3.5.1.6) has opened a new route for the hydantoinase process, suggesting that the enzyme, due to its broad substrate spectrum, can be used to obtain not only L‐α‐ but also L‐β‐, L‐γ‐ and L‐δ‐amino acids [1], thus opening new application opportunities for an old enzyme. NCβAA is also able to hydrolyse nonsubstituted substrate analogues in which the carboxyl group is replaced by a sulfonic or phosphonic acid group [18]. However, very little biochemical or structural information is available for this enzyme and only four prokaryotic β‐ureidopropionase enzymes have been characterised to date [18, 19, 20, 21].

N‐carbamoyl‐β‐alanine amidohydrolase, also known as β‐alanine synthase/β‐ureidopropionase, is the third enzyme participating in the degradation of uracil and thymine, which converts N‐carbamoyl‐β‐alanine and 2‐methyl‐N‐carbamoyl‐β‐alanine to β‐alanine and 2‐methyl‐β‐alanine, respectively [22]. The structure/function relationships for eukaryotic versions of these enzymes have been determined [23, 24]. Despite the same function, the prokaryotic versions of these enzymes are structurally and functionally more closely related to bacterial N‐L‐carbamoylases [18]. There are unpublished crystal structures of amidohydrolases from *Burkholderia* species in the PDB and of the L‐N‐carbamoylase from *Geobacillus stearothermophilus* CECT43, which has only 36% amino acid sequence identity to RrCβAA. In this study, we present the crystal structure of the *Rhizobium radiobacter* MDC 8606 N‐carbamoyl‐β‐alanine amidohydrolase, assess its activity profile at different experimental conditions and determine its activity against a range of substrates. Our findings illuminate key specificity features compared with L‐N‐carbamoylases, which show activity towards only N‐carbamoyl‐α‐amino acids. Our findings highlight the utility of this enzyme for a range of industrially relevant biotransformations for producing valuable amino acid products.

## Results and Discussion

### Analysis of the *R. radiobacter* MDC 8606 CβAA protein sequence

The gene encoding *R. radiobacter* MDC 8606 CβAA (Rr CβAA) was amplified from the DNA of a strain held in the Microbial Depository Centre (MDC) of the SPC Armbiotechnology NAS RA, Armenia. Analysis of the translated protein sequence confirms that this protein is a member of the carbamoyl‐amidohydrolase family (EC3.5.1.6; Fig. S1A), with between 20% and 97% amino acid sequence identity with enzymes in this family with demonstrated amidohydrolase activity. A phylogenetic tree depicting the relationship of these sequences is shown in Fig. S1B. Based on analysis of the sequence–activity relationships in this family and on the high degree of amino acid conservation in functionally important sites between the RrCβAA and bacterial N‐carbamoyl‐β‐alanine amidohydrolases, we propose this enzyme as a bacterial L‐N‐carbamoylase in the peptidase M20 family [1].

### Production and purification of recombinant *R. radiobacter* MDC 8606 CβAA

**Fig. 1:**
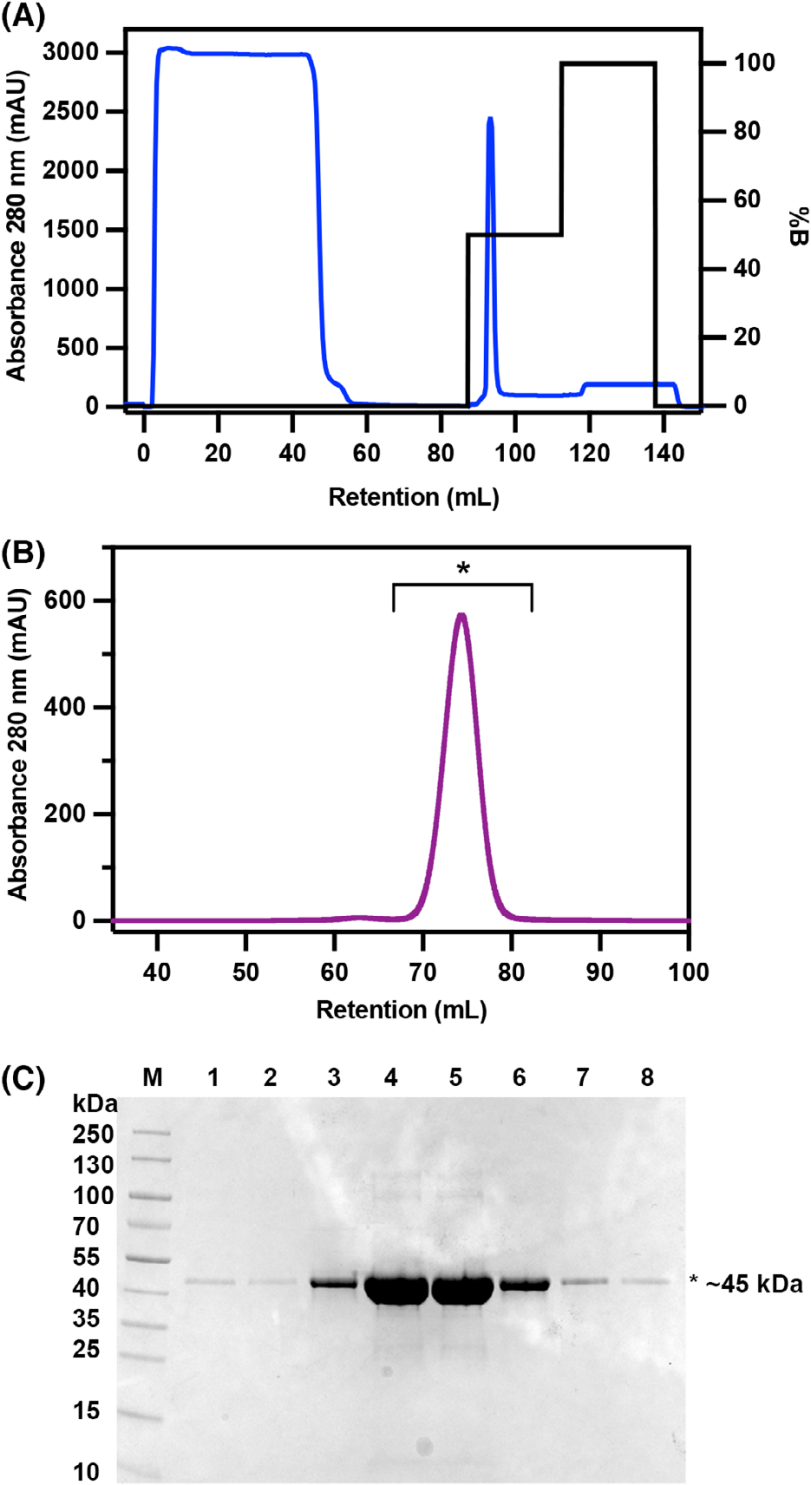
Purification of recombinant RrCβAA. (A) Chromatogram of RrCβAA purification by immobilised metal affinity chromatography. The sharp peak at 95 mL corresponds to the RrCβAA protein. (B) Recombinant RrCβAA was purified by size exclusion chromatography after immobilised metal ion chromatography. The sample was run on a Superdex S200 16/60 column equilibrated with buffer containing 50 mm Tris–HCl pH 8.0, 150 mm NaCl. A single major peak at 74.4 mL is visible on the chromatogram. Peak fractions between 62 and 86 mL (labelled with a star) were collected for downstream analysis. (C) SDS/PAGE of peak fractions (lanes 1–8) from the size exclusion chromatography run. The Fermentas prestained PageRuler was used as the molecular weight marker, and the gel was stained with Coomassie brilliant blue stain. The results shown in this figure are representative of three purification runs from individual *E. coli* BL21 colonies.

To study the biochemical and structural properties of the RrCβAA protein, a plasmid was assembled to produce a C‐terminally hexahistidine‐tagged recombinant version in *Escherichia coli* BL21(DE3). The protein was purified to homogeneity by a two‐step purification procedure, using immobilised metal affinity chromatography (Fig. 1A) and size exclusion chromatography (Fig. 1B). A single major peak was apparent on the size exclusion chromatogram at 74.4 mL. Based on the calibration of this column, this peak can be ascribed to a protein with an apparent molecular weight of 90 kDa, consistent with the protein being a dimer in solution. (Fig. 1B). SDS/PAGE analysis shows a main band of around 45 kDa, which is consistent with the calculated molecular weight of the protein of 44.7 kDa (Fig. 1C).

The purified protein from the 74.4 mL size exclusion fraction was 30 times more active against N‐carbamoyl‐β‐alanine than the crude lysate and displayed an activity of around 13.4 U·mg^−1^ under our standard assay conditions with the N‐carbamoyl‐L‐β‐alanine substrate (Table S1).

### *R. radiobacter* CβAA displays optimal activity between 50 and 60 °C

**Fig. 2:**
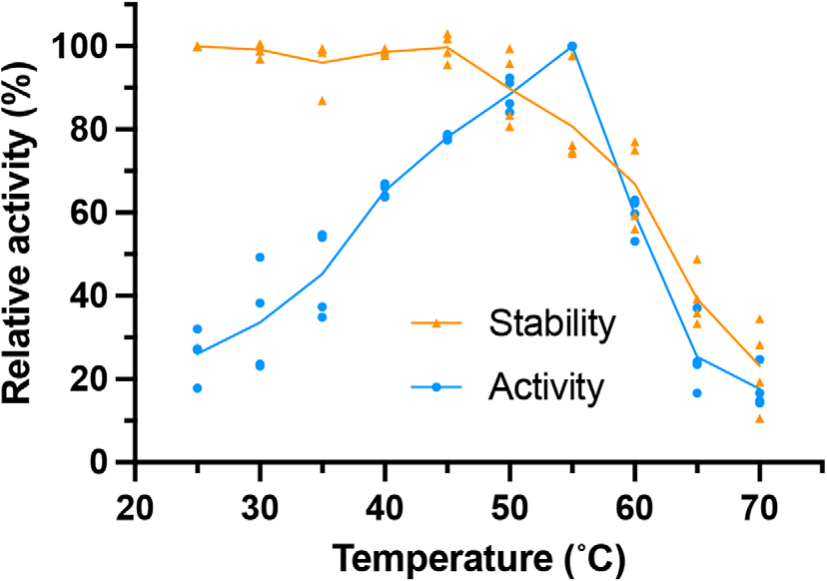
Activity and stability of RrCβAA with varying temperature. The activity and stability of the recombinant RrCβAA enzyme was assessed between 25 and 70 °C. Experiments were performed with two technical replicates each from two biological replicates. Blue points show the activity profile over the temperature range at a 15‐min end point; the blue line represents the mean of the four measured replicates. Orange points show residual activity of enzyme after 15‐min preincubation over the temperature range, prior to assay for 15 min at 40 °C; the orange line represents the mean of the four measured replicates.

To assess the impact of temperature on the activity profile of RrCβAA, the purified enzyme was assayed at temperatures between 25 and 70 °C for reaction times of 15 min. The normalised reaction progress data show a temperature optimum of 55 °C for the enzyme in the conditions tested (Fig. 2 blue line and Table S2). The thermostability of the enzyme was determined by incubating RrCβAA at different temperatures for 15 min and assessing the residual activity at 40 °C. The enzyme displayed no significant reduction in activity up to 40 °C, with 50% of activity lost at 65 °C (Fig. 2 orange line and Table S3).

### Divalent cations are required for Rr CβAA activity

**Fig. 3:**
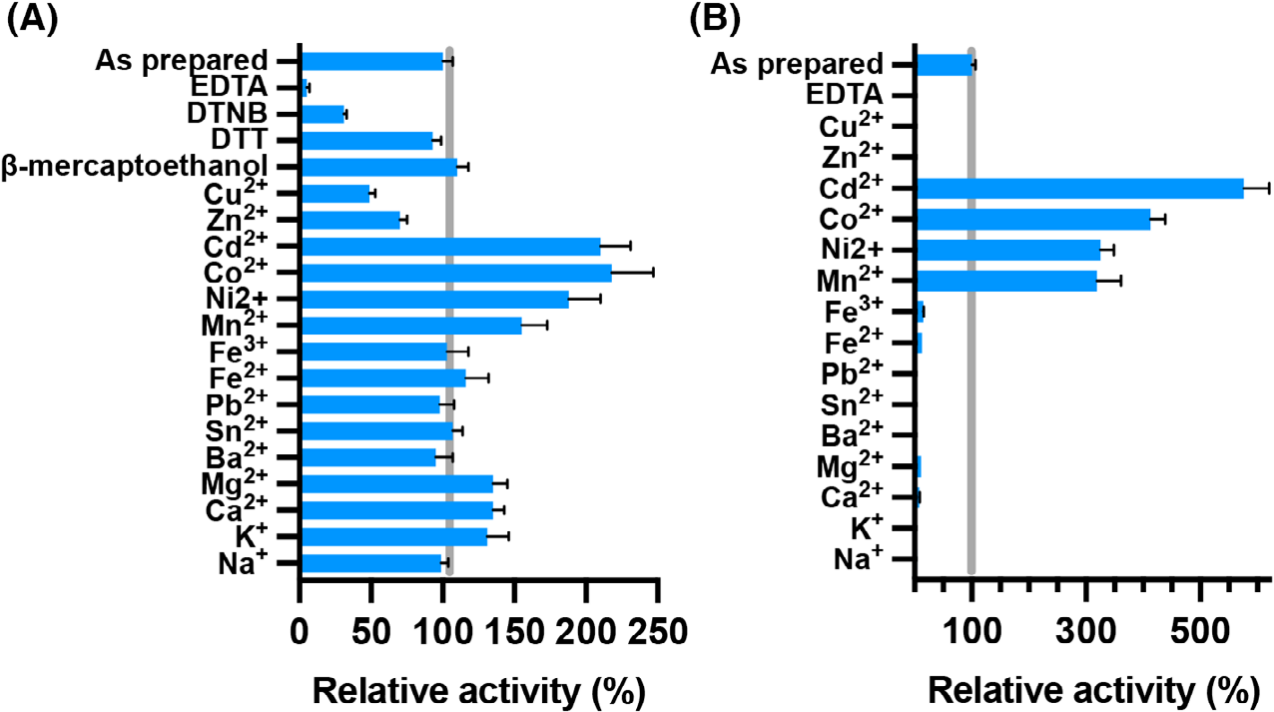
Effect of cations and chemical compounds on the activity of RrCβAA. (A) The activity of the recombinant RrCβAA enzyme was assessed after 1‐h incubation at 4 °C in the presence of 2 mm of different metals and EDTA, or 5 mm of DTNB, DTT and β‐mercaptoethanol. (B) The recombinant RrCβAA enzyme was incubated with EDTA prior to the addition of different metals. Experiments were performed with three technical replicates each from two biological replicates; error bars shown indicate the standard deviation from the mean of the replicate values. A specific activity of 13.6 U·mg^−1^ obtained without additives was defined as 100% activity; this is depicted as a grey line on the plots for reference. Error bars indicate standard deviations from the mean of the replicates.

The activity of the peptidase M20/M25/M40 family is known to be dependent on the presence of divalent cations in the active site to activate a catalytic water [25]. The purified RrCβAA enzyme was incubated in phosphate buffer with the addition of various cations, chelators and reducing agents (Fig. 3A and Table S4). The addition of EDTA abolishes almost all enzyme activity, which is consistent with the requirement of metal cations for enzyme activity. The enzyme showed only 5% of its original, as purified, activity after 1‐h incubation in the presence of 2 mm EDTA, whereas a continued overnight incubation fully inactivated RrCβAA. Assay of the purified protein with the addition of divalent cations showed that Cd^2+^, Co^2+^, Ni^2+^ and Mn^2+^ had a strong positive effect on the enzyme activity, while Zn^2+^ and Cu^2+^ have distinct inhibitory effects on the purified enzyme.

In our experiments, full activity recovery of EDTA‐inactivated enzyme was detected after incubation for 1 h at 4 °C in phosphate buffer containing Mn^2+^, Ni^2+^, Co^2+^ or Cd^2+^ at 2 mm concentration (Fig. 3B and Table S5). While eukaryotic N‐carbamoyl‐β‐alanine amidohydrolase has been described as a Zn^2+^‐dependent enzyme, our results show that the EDTA‐inactivated RrCβAA is not recovered with Zn^2+^. Moreover, addition of Zn^2+^ to the purified recombinant enzyme led to an inhibitory effect, whereas Cu^2+^ was shown to be a stronger inhibitor. Enzyme activity was not affected by Fe^2+^, which is known as an L‐N‐carbamoylase activator [26], nor by Sn^2+^ and Pb^2+^, known as inhibitors of *P. putida* IF0 12996 β‐ureidopropionase [20]. Similar results were seen for βcar_At_ from *Agrobacterium tumefaciens* C58 [18], where the enzyme activity can be recovered with Mn^2+^, Ni^2+^ and Co^2+^. Interestingly, the activity of βcar_At_ could not be recovered with Cd^2+^, which is one of the preferred metal cations for RrCβAA. Moreover, Cd^2+^ shows an inhibitory effect on β‐ureidopropionase from *Pseudomonas putida* IFO 12996 [20].

ICP‐MS analysis of the purified recombinant protein from *E. coli* indicated that the protein is loaded with 22 ± 3% of Zn^2+^, 10 ± 1% of Mn^2+^ and 6.5 ± 0.7% of Ni^2+^. Only trace amounts (< 1%) of Co^2+^, Cu^2+^ and Cd^2+^ were detected. While these data suggest that RrCβAA is a Zn‐dependent enzyme, metalloproteins produced in heterologous expression hosts can become assembled with the wrong metal because of mismatches between the free energies for protein metalation and the metal availabilities in the expression host [27]. Thus, it is not possible to ascertain the identity of the physiological metal cofactor (the metal cofactor used by the enzyme in *R. radiobacter* cells) from these experiments. Nevertheless, given the demonstrated inhibition of the enzyme by additional Zn^2+^ and activation by additional Mn^2+^ or Ni^2+^, it is likely that the latter metals are the relevant cofactors. We note that the bound metals are bound stably, as they were retained following elution of the protein through a desalting column. However, these bound metals are likely exchangeable, as the enzyme is readily inactivated by EDTA treatment and reactivated by addition of excess metals *in vitro*.

Disulfide reducing agents such as β‐mercaptoethanol and DTT do not show any inhibitory effects on the activity of the enzyme. Enzyme activity was not altered in the presence of 2 and 5 mm β‐mercaptoethanol. Interestingly, the sulfhydryl reagent DTNB showed an inactivating effect on the enzyme. βcar_At_ from *Agrobacterium tumefaciens* C58 was not inhibited by DTNB [18], whereas this compound showed inhibitory effect on other β‐ureidopropionase and L‐N‐carbamoylase proteins. The catalytic domain has a number of cysteine residues, including Cys364, which is within 10 Å of the active site and close to the interdomain hinge. This residue is only partially conserved in other members of the enzyme family (Fig. S1A), which may explain the different inhibitory effects seen. Our results indicate that while cysteine residues do not play a key role in the enzyme activity, DTNB may form a covalent adduct that interferes with the activity of the enzyme through allosteric effects, or by interfering with the folding and stability of the protein.

### RrCβAA displays a broad substrate range with optimal activity against N‐carbamoyl‐L‐β‐alanine

**Fig. 4:**
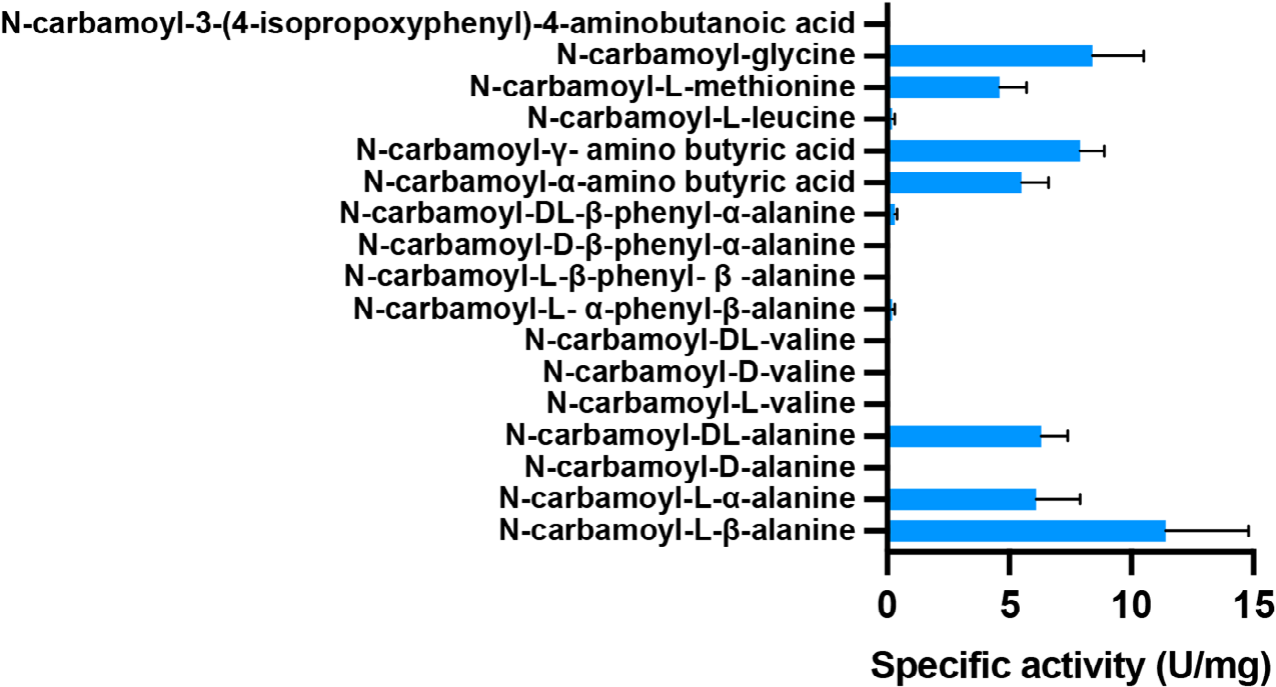
Substrate specificity of RrCβAA. The specific activity of purified RrCβAA was assessed towards various N‐carbamoyl‐amino acids. A reaction mixture containing 100 mm of different N‐carbamoyl‐amino acids was incubated at 40 °C for 10 min. The reaction was started by adding enzyme and was carried out for 15 min at 40 °C, pH 8.0. Experiments were performed with two technical replicates each from two biological replicates; error bars shown indicate the standard deviation from the mean of the replicate values.

To better understand the substrate specificity of the RrCβAA enzyme, we investigated its activity against N‐carbamoyl‐L‐, D‐ and DL‐ amino acids (Fig. 4 and Table S6). The enzyme displays no activity towards N‐carbamoyl‐D‐amino acids, with a clear stereo specificity towards N‐carbamoyl‐L‐amino acids. Consistent with the function of this enzyme in catalysing the third step in the pyrimidine degradation pathway, it shows the greatest catalytic efficiency for N‐carbamoyl‐L‐β‐alanine. This is in contrast with the recombinant Atβcar from *Agrobacterium tumefaciens* C58, which displayed the highest activity towards N‐carbamoyl‐L‐methionine [28]. The RrCβAA enzyme displayed a specific activity towards N‐carbamoyl‐L‐α‐alanine that was twofold lower than to N‐carbamoyl‐L‐β‐alanine, which differs only by the position of the carbamide group. In the case of N‐carbamoyl‐L‐β‐alanine, the carbamide group is located at the edge of the β‐carbon position, resulting in a linear structure. Similar results have been obtained with N‐carbamoyl‐α‐amino‐ and N‐carbamoyl‐γ‐amino butyric acids. The movement of the carbamide group from α‐ to the γ‐position resulted in a more than 1.5‐fold increase in the specific activity. Conversely, RrCβAA displayed very low activity against N‐carbamoyl‐L‐valine and N‐carbamoyl‐L‐leucine. Similar results were obtained with N‐carbamoyl‐L‐α‐phenyl‐β‐alanine and N‐carbamoyl‐L‐β‐phenyl‐β‐alanine. RrCβAA displayed good activity towards N‐carbamoyl‐L‐methionine, which implies that the sulfur‐containing sidechain can be accommodated within the active site.

Our results indicate that RrCβAA has a distinct preference for N‐carbamoyl‐L‐amino acids with linear R‐groups and that its active site does not readily accommodate branched hydrophobic, or aromatic sidechains.

### Crystal structure RrCβAA

**Fig. 5:**
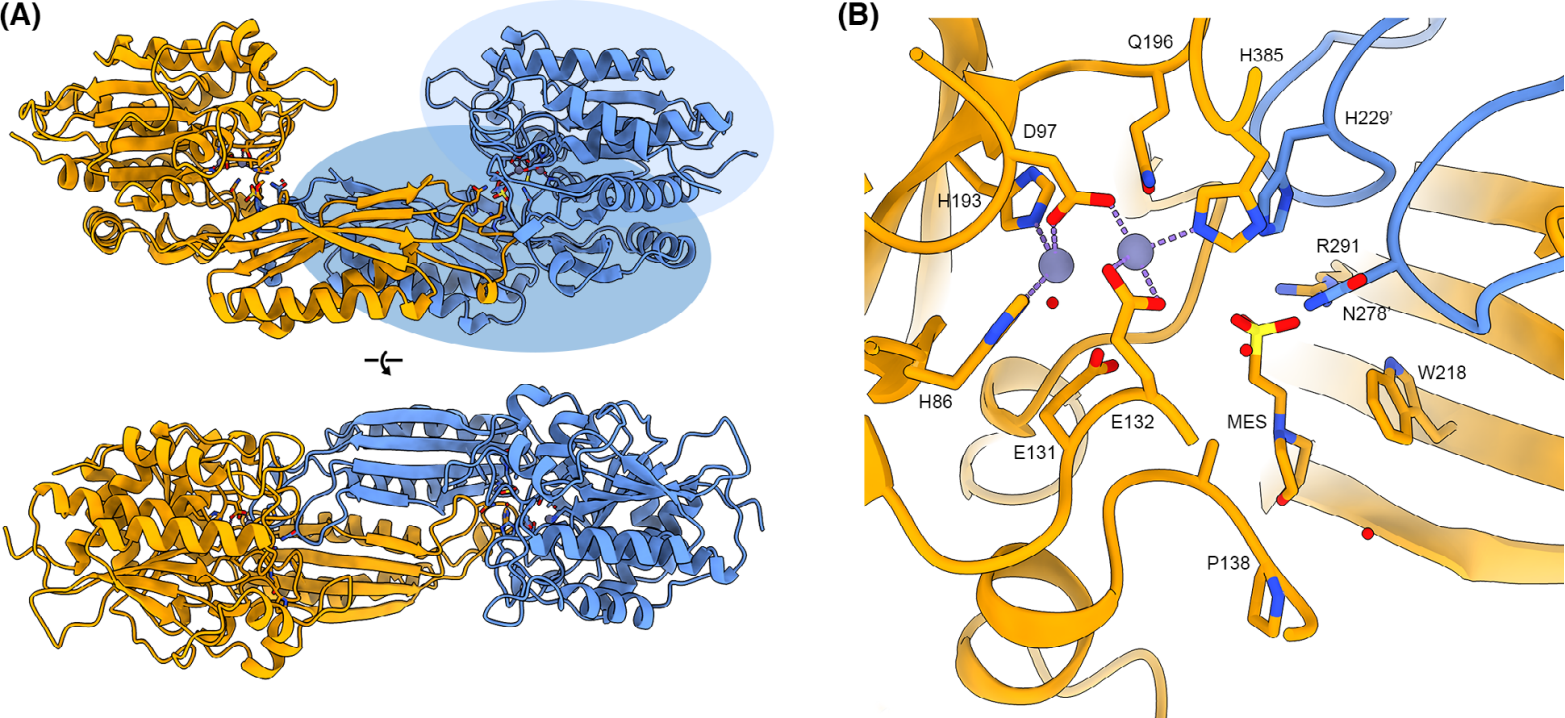
Crystal structure of RrCβAA. (A) Overall structure of RrCβAA shown in cartoon depiction, monomers are coloured orange and blue. The dimerisation domain of one monomer is highlighted in mid‐blue, with the catalytic domain shown in light blue. (B) Metal and ligand binding site of RrCβAA with interacting residues shown in stick representation coloured by atom. The ligand binding site comprises residues from both monomers, shown in orange and blue. Zinc ions are shown as purple spheres with coordinating bonds shown as purple dashes. A competing MES buffer ligand molecule from the crystallisation condition is bound in the ligand binding site. Figure prepared using chimerax version 1.6.1.

Based on our results demonstrating a requirement for metal binding for catalysis and our exploration of the substrate preference of the RrCβAA, we determined the crystal structure of RrCβAA to better understand the structure/function relationships that mediate substrate specificity in this enzyme. The structure of RrCβAA was determined to 2 Å resolution in the P22121 space group, with two molecules in the asymmetric unit representing the functional dimer of the protein (Table 1; Fig. 5A). The RrCβAA enzyme has a discontinuous catalytic domain from the N terminus to residue 213 and residue 331 to the C terminus. The dimerisation domain is intercalated within the catalytic domain and comprises residues 214–330. The dimerisation interface is formed between beta‐strands on one face of the domain (residues 269–278) and alpha helices on the opposite face (residues 230–260). The interface has a hydrophobic core, formed by the side chains of residues from both the strands and helices, a network of hydrogen bonds stabilising the beta‐strand interface, and salt bridges across the top face of the alpha‐helical interface (Fig. S2).

**Table 1:** X‐ray crystallographic data collection and refinement statistics.

Data collection and analysis statistics	
Beamline	DLS I04
Date	15/12/2019
Wavelength (Å)	0.912
Resolution (Å)	84.92–2.00 (2.05–2.00)
Space group	P2_1_22_1_
Unit‐cell parameters	
*a* (Å)	53.23
*b* (Å)	104.62
*c* (Å)	145.37
α = β = γ (°)	90.00
Unit‐cell volume (Å^3^)	809 598
Solvent content (%)	46
Measured reflections	391 722 (29 077)
Independent reflections	55 817 (4087)
Completeness (%)	100.0 (100.0)
Redundancy	7.0 (7.1)
CC_1/2_ (%)	0.996 (0.786)
<I>/<σ(I)>	7.1 (1.3)
Model refinement	
Rwork (%)	22.87
Rfree (%)	26.98
No. of non‐H atoms	
Protein	6220
Solvent	362
Ions	4
RMS deviation from ideal values	
Bond angles (°)	1.85
Bond lengths (Å)	0.013
Average B‐factors (Å^2^)	
Protein	31.7
Solvent	32.0
Ions	35.0
Ramachandran plot	
Most favoured regions (%)	98
PDBID	8C46

^a^ Values for highest resolution shell are shown in parentheses.

The catalytic and dimerisation domains of RrCβAA are attached through a flexible hinge region at the domain interface. In other structural models of proteins in this family, the catalytic domain rotates around this hinge to open and close the active site cleft. When aligned to other models, it is apparent that our RrCβAA model is in a partially closed state, with the catalytic domains of both chains in the asymmetric unit adopting essentially identical conformations. The hinging movement shown in previously determined models is found on a continuum between fully closed [29] and a wide‐open state [30] (Fig. S3A). The models show a rotation range of approximately 45°; and when the catalytic domains are aligned, there is a relative 30 Å movement around the axis of rotation between the closed and open states at the end of the dimerisation domain (Fig. S3B).

Each protein chain in the dimer has electron density features consistent with the presence of divalent cations in the putative metal binding site. In the absence of X‐ray anomalous scattering data, the cations were iteratively modelled with varying degrees of occupancy by Mn^2+^ and Zn^2+^. The final model contains four Zn^2+^ cofactors per dimer. Based on our assays and ICP‐MS data, we do not consider Zn^2+^ to be the physiological metal cofactor. Instead, we propose that the protein has become inserted with available Zn^2+^ ions during the process of recombinant production, purification and crystallisation. The modelled metal ions coordinate conserved glutamic acid and histidine residues, with ligand coordination distances of approximately 2.1 Å (Fig. 5B). A strong peak of electron density was observed in the vicinity of the active site, and a MES buffer molecule from the crystallisation condition was modelled in this region. The modelled MES refined well with good electron density fit and B‐factors (Fig. 5B and Fig. S4).

**Fig. 6:**
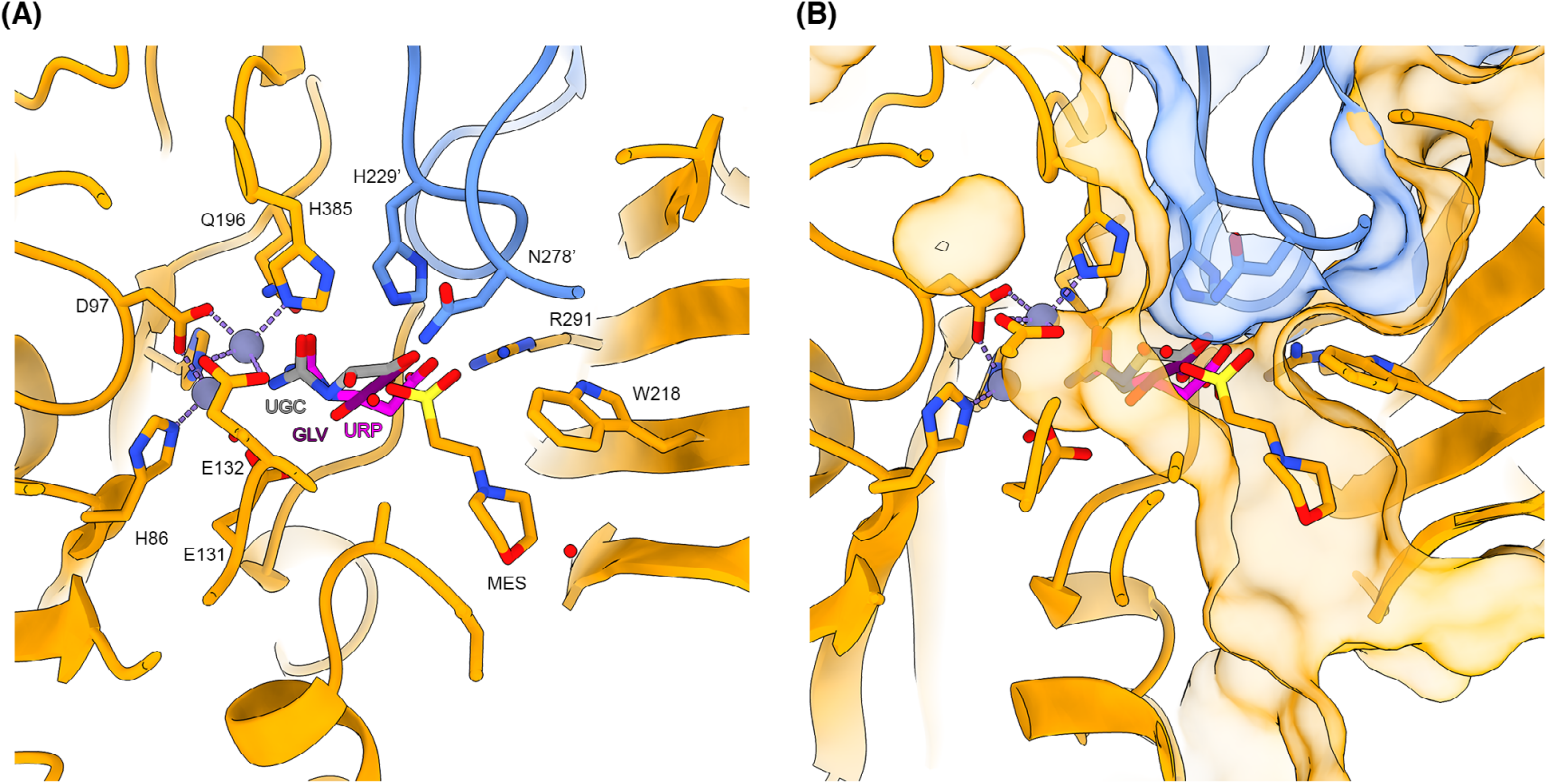
Productive ligand binding in RrCβAA is constrained by a tight active site cleft in the closed conformation. (A) Structural homologues with bound ligands were aligned to the RrCβAA structural model. RrCβAA is shown as orange and blue cartoons with metal and ligand binding residues shown as stick representations with bound MES buffer shown. Modelled ligands are as follows: UGC—(S)‐ureidoglycolate from PDB: 4PXB; GLV—β‐alanine from PDB 2V8G; URP—N‐carbamoyl‐β‐alanine from PDB: 5THW. (B) Active site cleft shown with transparent surface to highlight the physical constraints placed on ligand binding in this space. Figure prepared using chimerax version 1.6.1.

The presence of high concentrations of the competing MES buffer in the crystallisation condition hindered experiments to soak ligands, such as N‐carbamoyl‐β‐alanine, into the active site of the crystals to determine a structure of an enzyme ligand complex. Structural alignments of our RrCβAA model with other structures in this family with ligands in their active sites give some insight into the ligand binding site (Fig. 6A).

The carbamoyl group of the modelled ligands occupies a space close to the metal binding site, where the group is oriented through interactions with the cations and cluster of conserved amino acids including glutamine (Gln196), glutamic acid (Glu131) and histidine (His385) residues. The carboxylic acid group of the ligand forms a salt bridge with the conserved arginine residue (Arg291). These interactions essentially constrain the ligand binding at both functional groups. To form a productive ligand complex, the protein must engage ligand while in an open state and close around it to facilitate catalysis [30]. The observed preference for linear and gamma‐substituted carbamoyl amino acids is a consequence of the steric constraints posed by the amino acids lining the active site cleft (Fig. 6B). Large aromatic carbamoyl amino acids cannot fit within the closed active site, and therefore, the enzyme is not active against them. However, linear side chains at the alpha and beta positions may be accommodated in the active site cleft to form productive complexes.

## Conclusion

In this work, we have demonstrated the recombinant production and activity of the *R. radiobacter* N‐carbamoyl‐β‐alanine amidohydrolase enzyme (RrCβAA). RrCβAA was purified as a homodimer, like other β‐ureidopropionase and L‐N‐carbamoylases except L‐N‐carbamoylases characterised from *Brevibacillus reuszeri* HSN1 and *Pseudomonas* sp. ON‐4, which have been shown to exist as a homotrimer and homotetramer, respectively [31]. Among studied carbamoylases with L‐stereospecificity, only N‐carbamoyl‐β‐alanine amidohydrolase of *A. tumefaciens* C58 and L‐N‐carbamoylase of *P. putida* IFO 12996 have been demonstrated to have β‐ureidopropionase activity (Table 2).

**Table 2:** Comparison of biochemical properties of L‐N‐carbamoylases with respect to N‐carbamoyl‐β‐alanine amidohydrolases from *Rhizobium radiobacter* MDC 8606. For activity with metal cofactors, blue shading represents activation and orange represents inhibition and blank cells show data not available.

Source	Specificity with Ureidopropionase substrate	Subunit mass (kDa)	Oligomer	Activity with metals										pH optimum	Temperature optimum (°C)	References
				Ca	Mn	Fe	Co	Ni	Cu	Zn	Ag	Cd	Hg			
*R. radiobacter* MDC 8606	Yes	45.0	Monomer											ND	55	This study
*B. reuszeri* HSN1	No	44.3	Trimer											8.5	50	[55]
*A. tumefaciens* C58 (β‐up)	Yes	45.0	Dimer											8	30	[18]
*A. xylosoxidans* AKU 990	ND	65.0	Dimer											8–8.3	30	[56]
*A. aurescens* DSM3747	No	44.0	Dimer											8.5	50	[32]
*B. kaustophilus* CCRC1123	No	45.0	ND											7.4	70	[57]
*P. putida* IFO 12996 (β‐up)	Yes	45.0	Dimer											7.5–8.2	60	[20]
*Pseudomonas* sp. NS671	No	45.0	Dimer											7.5	40	[33]
*B. stearothermophilus* NSl122A	ND	ND	ND											8	60–70	[34]
*G. stearothermophilus* CECT43	ND	ND	ND											7.5	65	[35]
*Pseudomonas* sp. ON‐4A	ND	45.0	Tetramer											9	50	[31]
*S. meliloti* CECT4114	No	42.0	Dimer											8.0	60	[26]

All β‐ureidopropionase and L‐N‐carbamoylases are described as metalloenzymes and RrCβAA is no exception to this rule. The chelating agent EDTA abolishes enzyme activity, which was recovered by the addition of Mn^2+^, Ni^2+^, Co^2+^ or Cd^2+^. The first three metals are well‐known cofactors for this enzyme family, while Cd^2+^ has not been widely demonstrated as a cofactor for this enzyme, and in some cases has been shown to be inhibitory [20]. This is the first result showing that Cd^2+^ can act as a cofactor for this class of enzymes, although it is not clear from our structural analysis what the molecular basis for the potential differences in metal preferences is. The other divalent cations tested, such as Cu^2+^ and Zn^2+^, show an inhibitory effect on enzyme activity. These results indicate that the properties of RrCβAA are comparable to those of other known L‐carbamoylases and β‐ureidopropionase enzymes from other bacterial strains (Table 2).

Reducing compounds did not show an inhibitory effect on enzyme activity, consistent with our structural observations showing that there are no key cysteine residues involved in the catalysis. The enzyme is also not stabilised by any disulfide bridges, which may be disrupted by reducing agents. The inhibitory effect of DTNB on the enzyme can be rationalised if it forms a covalent adduct with Cys364, which is close to the hinge region of the protein. Such an adduct would prevent closure of the active site and inhibit the production of a catalytically competent intermediate state with any substrate.

The optimum activity for the RrCβAA was recorded at 55 °C, which is higher than the enzymes from *Agrobacterium tumefaciens* C58 [28], *Arthrobacter aurescens* DSM3747 [32], *Achromobacter xylosoxidans* [20] and *Pseudomonas* sp. NS671 [33], but slightly lower than the enzymes from *P. putida* IFO12996 [20], *Bacillus stearothermophilus* NSl122A [34] and *Geobacillus stearothermophilus* CECT43 [35].

In terms of substrate preference and promiscuity, the RrCβAA shows good activity against L‐α‐, L‐β‐, L‐γ‐amino acids. This contrasts with other L‐carbamoylases described so far, which show preferential activity to only N‐carbamoyl L‐α‐amino acids. The lack of activity towards branched chain and aromatic amino acids limits its use against these substrates. However, there is certainly scope for employing focused mutagenesis to open the substrate binding site to accept these substrates. This strategy has been as demonstrated for the *Sinorhizobium meliloti* carbamoylase, which has been engineered to accept aromatic amino acids [29]. Further work on the RrCβAA enzyme will focus on expanding its substrate scope against these aromatic amino acids with high potential for use in industrially useful chemo‐enzymatic cascades.

## Materials and methods

### Reagents and substrates

Phusion® DNA Polymerase, BsaI restriction enzyme and T4 DNA ligase were purchased from New England Biolabs (Hitchin, UK). Isopropyl β‐D‐1‐thiogalactopyranoside (IPTG) was purchased from Merck, UK (Gillingham, Dorset, UK). The molecular weight marker for SDS/PAGE was purchased from Thermo Fisher Scientific (Cramlington, UK). Standards and some substrates (N‐carbamoyl‐β‐alanine (3‐ureidopropionic acid), N‐carbamoyl‐glycine) were purchased from Sigma. Other N‐carbamoyl‐DL, L and D‐amino acids have been synthesised for this study. ^1^H and ^13^C NMR analyses were performed to confirm their structures (Fig. S6). All other chemicals were of analytical grade.

### Bacterial strains and plasmids

The *Rhizobium radiobacter* MDC 8606 strain used as a source for the N‐carbamoyl‐β‐alanine amidohydrolase (RrCβAA) gene was taken from the Microbial Depository Center (MDC) of SPC ‘Armbiotechnology’ NAS RA. *Escherichia coli* Top 10 and *E. coli* BL21 (DE3) strains were used for propagation of plasmids and protein expression, respectively. A modified pET28 plasmid for Golden Gate cloning was a gift of Dr Laura Tuck.

### Nucleotide and amino acid sequence analysis

Sequence analysis of the RrCβAA gene was performed using the BLAST program [36]. Protein sequence alignments were performed in Multalin [37] and figures prepared with ESPript [38]. The nucleotide sequence data of the isolated RrCβAA, as well as 16s rRNA genes of *Rhizobium radiobacter* MDC 8606 strain, were deposited in NCBI GeneBank database with the accession numbers MT542139 and MT534525, respectively.

### Cloning, expression and purification of RrCβAA

To amplify the *R. radiobacter* MDC 8606 N‐carbamoyl‐β‐alanine amidohydrolase open reading frame, primers RrCβAA‐F (5′**GACGGTCTCTA**ATGACGGCGGGTAAAAACTTGAC3′) and RrCβAA‐R (5′**GACGGTCTCTACCT**TTGCACGATCTCCGCAGTCTC3′) were designed using *Agrobacterium tumefaciens* C58 N‐carbamoyl‐β‐alanine amidohydrolase gene sequence as a template (GenBank: EF507843.1). PCR was performed using these primers against the purified *R. radiobacter* MDC 8606 genomic DNA with the following conditions: 98 °C for 1 min, followed by 30 cycles of 98 °C for 30 s, 60 °C for 30 s and 72 °C for 1 min, followed by a final elongation at 72 °C for 10 min. After examination by 1% agarose TAE electrophoresis, the amplified product was purified by QIAquick PCR Purification Kit. The purified DNA fragment was then assembled via one‐pot Golden Gate cloning [39] into a CIDAR MoClo [40] compatible pET28 vector via BsaI restriction sites introduced into the PCR product and pET28 vector. The resulting ligation product was transformed into chemically competent *E. coli* TOP10 cells with selection on Lysogeny Broth (LB) agar plates supplemented with 35 μg·mL^−1^ kanamycin, 1 mm IPTG and 20 μg·mL^−1^ X‐Gal. Recombinant plasmid was extracted from white insert‐positive clones by miniprep using a Qiagen Miniprep kit. The insert presence was confirmed by Sanger sequencing of the purified plasmids. The sequence‐verified plasmid was transformed into *E. coli* BL21(DE3) cells with selection on LB agar supplemented with 35 μg·mL^−1^ kanamycin. A single colony was grown overnight at 37 °C in 100 mL LB medium, supplemented with 35 μg·mL^−1^ kanamycin, with shaking at 180 rpm. The cells were subcultured into 2 L of LB, grown until OD_600_ 0.5, and recombinant protein production was induced with 1 mm IPTG, at 25 °C, followed by incubation for a further 16 h.

Cells were harvested by centrifugation at 7000 ***g*** for 20 min. The harvested cells were resuspended in 10× w/v Buffer HisA (50 mm imidazole, 500 mm NaCl, 50 mm Tris–HCl, pH 8.0) and subsequently sonicated on ice for 5 min with 30 s on/off cycles at 60 watts power output. The lysate was cleared by centrifugation at 35 000 ***g*** and filtered with a 0.45 μm syringe filter.

Cell‐free extract was applied to a 5 mL HisTrap FF column (GE Healthcare), and unbound proteins were washed off with 10 column volumes of Buffer HisA (50 mm Tris–HCl, pH 8.0, 500 mm NaCl, 50 mm imidazole). A step gradient of 50% and 100% Buffer HisB (50 mm Tris–HCl, pH 8.0, 500 mm NaCl, 500 mm imidazole) was used to elute His‐tagged proteins. Fractions of HisTrap eluent containing the protein of interest were pooled and concentrated by Vivaspin Turbo (Sartorius, 10 kDa MWCO) centrifugation devices at 4000 ***g***, 18 °C. The concentrated protein was then subjected to size exclusion chromatography using an S200 16/60 column (Cytiva), equilibrated with Buffer GF (50 mm Tris–HCl, pH 8.0, 150 mm NaCl). Calibration data for the S200 16/60 gel filtration column used are available at https://doi.org/10.6084/m9.figshare.7752320.v1. Fractions were analysed by sodium dodecyl sulfate polyacrylamide gel electrophoresis using Mini‐PROTEAN TGX precast 4–20% gels (BioRad) according to the standard method [41] to determine the molecular weight and the purity of the samples. Purified RrCβAA was concentrated and analysed by SDS/PAGE, after incubating the sample for 5 min at 95 °C temperature, in the presence of 5 mm β‐mercaptoethanol. The Fermentas prestained PageRuler was used as a protein molecular weight marker for SDS/PAGE. Gels were stained with Coomassie brilliant blue for visualisation of protein bands.

For characterisation studies, purified RrCβAA was placed into 100 mm phosphate buffer, pH 8.0 (Tris–HCl shows absorption in the presence of the ortho‐phthalaldehyde reagent) and stored at −80 °C with the addition of 50% (v/v) glycerol for enzyme characterisation.

### General procedure for synthesis of carbamoyl amino acids

All chemicals used for synthesis were of analytical or reagent grade. N‐Carbamoyl‐β‐Ala (**15**) was from ‘Sigma’. The compounds 2–14 were prepared using the amino acids from Reanal (Budapest, Hungary). All compounds were recrystallised to purify from starting materials, and melting points were determined on a Boetius PHMK 76/0904 hot‐stage microscope (GDR) and were uncorrected. ^1^H and ^13^C NMR spectra were recorded on a Varian Mercury‐300 spectrometer, operating at 300 MHz; chemical shifts are reported in *δ* values (ppm) relative to tetramethylsilane as internal standard. Coupling constants (*J* values) are given in Hertz (Hz). The solvents mixture was DMSO‐d_6_/CCl_4_; NMR spectra and assignments are shown in the Supplementary Information (Fig. S5); the signals are reported as follows: s (singlet), d (doublet), t (triplet), q (quartet), dd (double doublet), p (pentet), sp (septet), m (multiplet), br. (broad).

The mixture of equimolar amounts of amino acid and sodium cyanate (NaOCN) in water was kept at a room temperature for 75–80 h (Compounds: **2**, **4**, **7**–**13**) or at 100 °C for 4 h (Compounds: **3**, **5**, **6**). Then, pH of reaction mixture was adjusted to 2–3 with concentrated HCl. The separated solid was filtered, washed with water, and recrystallised. From filtrate, additional amount of product was obtained after concentrating at reduced pressure. Reaction **14** was carried out in 75% ethanol (100 °C, 4 h). After removing ethanol under reduced pressure, water was added, and pH was adjusted to 5–6 with concentrated HCl. The separated product was treated as above.

### RrCβAA activity assay

RrCβAA assays were performed at 40 °C. The reaction mixture contained 100 mm phosphate buffer (pH 8.0) and 5 μg purified enzyme in a total volume of 0.1 mL. Reactions were started by the addition of 0.1 mL N‐carbamoyl‐L‐β‐alanine to final concentrations of 100 mm after preincubation of both reaction mixture and substrate solutions at 40 °C for 10 min. After 15 min, the reaction was stopped by adding 30% w/v trichloroacetic acid (TCA) to a final concentration of 3% w/v. Specific activity of RrCβAA was determined using an assay able to detect β‐alanine concentration, upon conversion into an isoindole derivative by reaction with ortho‐phthalaldehyde (OPA) [42]. Particular attention was paid to the OPA reaction conditions, as it has been reported that the derivative of β‐alanine is unstable. For this reason, a high concentration of reagents (20 times excess of OPA and 50 times excess of β‐mercaptoethanol compared with the β‐alanine product) was used to stabilise the final product. Thus, 3 mL of freshly prepared activity reagent (0.1 m sodium borate pH 9.6, 2.5 mm OPA and 2.5 mm β‐mercaptoethanol) was added to each sample, followed by incubation at 20 °C for 30 min. β‐alanine concentrations were determined spectrophotometrically at SF‐46 (‘Lomo’, Russia) based on the absorption of the corresponding isoindole at 340 nm. The extinction coefficient for each substrate was calculated separately (extinction coefficient data are available in Fig. S6. For preparation of standard curves, 40 mm concentration of L‐β‐alanine, L‐α‐alanine, L‐α‐valine, L‐β‐phenyl‐α‐alanine, α‐amino butyric acid, γ‐amino butyric acid, L‐α‐leucine, L‐α‐methionine and glycine was prepared and the adsorption of serial dilutions of amino acids at final concentrations of 0.032, 0.064, 0.096, 0.128 and 0.16 mm was measured. One unit of enzyme activity was defined as the amount of enzyme catalysing the formation of one micromole of product per minute under the above‐mentioned conditions. Specific activity was calculated per milligram of protein. All measurements were done at least in two separate experiments with two replicates. Data files supporting these analyses are available at https://doi.org/10.6084/m9.figshare.24025392.v1.

### RrCβAA temperature optimum and thermostability

For determination of the optimal temperature for RrCβAA, enzymatic activity was measured under the described conditions at various temperatures ranging from 25 to 70 °C. Thermostability of purified RrCβAA was investigated by incubating RrCβAA at various temperatures (25–70 °C) for 15 min in phosphate buffer, followed by incubation on ice. Residual activities were determined under the above assay conditions.

### Effect of metals and chelation on RrCβAA activity

Metal ions are generally considered as important factors affecting microbial enzyme activity. The effects of various mono‐ and bivalent metal ions (including NaCl, KCl, CaCl_2_, MgSO_4_, BaCl_2_, SnCl_2_, PbSO_4_, FeCl_3_, FeSO_4_, CuSO_4_, ZnSO_4_, MnSO_4_, CdCl_2_, NiCl_2_ and CoSO_4_) and chemical compounds (including EDTA, 5,5′‐dithiobis‐(2‐nitrobenzoic acid) (DTNB), dithiothreitol (DTT) and β‐mercaptoethanol) on RrCβAA activity was investigated. Metal salts were freshly prepared for each assay, and iron salts were prepared with freshly degassed deionised water and used immediately. RrCβAA was incubated in the presence of 2 mm of each metal ion, DTT and EDTA, or 5 mm of DTNB and β‐mercaptoethanol, for 1 h at 4 °C. A control was performed in the absence of any tested compound. To test recovery of enzyme activity after metal removal, the enzyme was incubated with 5 mm EDTA at 4 °C for 1 h to chelate metals and then dialysed against excess reaction buffer containing 2 mm of each metal ion tested. All the activity assays were performed in triplicate.

### Substrate spectrum and enantioselectivity of RrCβAA

The specific activity of purified RrCβAA towards various N‐carbamoyl‐amino acids including N‐carbamoyl‐L‐β‐alanine, N‐carbamoyl‐L‐α‐alanine, N‐carbamoyl‐D‐α‐alanine, N‐carbamoyl‐DL‐α‐alanine, N‐carbamoyl‐L‐α‐valine, N‐carbamoyl‐D‐α‐valine, N‐carbamoyl‐DL‐α‐valine, N‐carbamoyl‐L‐β‐phenyl‐α‐alanine, N‐carbamoyl‐L‐β‐phenyl‐β‐alanine, N‐carbamoyl‐D‐β‐phenyl‐α‐alanine, N‐carbamoyl‐DL‐β‐phenyl‐α‐alanine, N‐carbamoyl‐α‐amino butyric acid, N‐carbamoyl‐γ‐amino butyric acid, N‐carbamoyl‐L‐α‐leucine, N‐carbamoyl‐L‐α‐methionine and N‐carbamoyl‐α‐glycine was measured using the above method. A calibration curve for each product was constructed, and extinction coefficient for each product has been calculated (Fig. S6). Neither the isoindole formed from ammonium ions, nor N‐carbamoyl‐amino acids gave a detectable signal under the chosen reaction conditions.

### Protein quantification

The concentration of the purified was determined by a colorimetric technique using the PierceTM BCA protein assay kit following manufacturer's specifications for the standard test‐tube procedure at 37 °C. Diluted bovine serum albumin (BSA) standards were prepared in GF buffer, and a calibration curve of absorbance at 562 nm against concentration was plotted (Fig. S7). Protein sample absorbance was measured at 562 nm (average of three experimental replicates), and the concentration was calculated.

### Protein crystallography

Purified recombinant RrCβAA was concentrated to 15 mg·mL^−1^ using a 10 kDa MWCO centrifugal concentrator (Vivaspin) and subjected to sitting drop vapour diffusion crystallisation screening with commercial screens from Molecular Dimensions and Hampton Research. Drops of 100 nL protein plus 100 nL well solution were set up against wells containing 70 μL of crystallisation solutions. After 2 weeks, crystals were found in row D of the PACT premier screen (Molecular Dimensions (Rotherham, UK)). An optimisation screen based on this condition was set up in 24‐well plates by varying the PEG1500 concentration and MMT buffer pH. Drops of 1 μL protein and 1 μL well solution were set up on plastic cover slips over wells containing 1 mL crystallisation solution. Crystals grew in a well solution containing 23% (w/v) PEG1500 and 100 mm MMT pH 6.0. Crystals were harvested with a LithoLoop (Molecular Dimensions Limited) and transferred to a cryoprotection solution of well solution supplemented with 50% (v/v) PEG400. Cryoprotected crystals were flash‐cooled in liquid nitrogen. Diffraction data were collected at Diamond Light Source; data collection and model refinement statistics are shown in Table 1. Diffraction data are available at doi:https://doi.org/10.5281/zenodo.7331274.

The dataset was integrated with XIA2 [43] using DIALS [44] and scaled with Aimless [45]. The space group was confirmed with Pointless [46]. The phase problem was solved with MorDa. Initial model building was performed with CCP4build task on CCPcloud [47]. The model was refined with iterative cycles of refmac [48], or BUSTER, intercalated with manual model building with COOT [49]. The model was validated using Coot and Molprobity [50]. Other software used were from CCP4 cloud and the CCP4 suite [51]. Structural figures were produced with chimerax version 1.6.1 [52].

### Inductively coupled plasma mass spectrometry analysis

RrCβAA protein was subjected to ICP‐MS analyses using Durham University Bio‐ICP‐MS Facility (PlasmaLab software; Thermo Fisher). The protein was analysed as purified and following elution from a desalting column to remove any loosely bound metals. Raw detected values for ^55^Mn, ^59^Co, ^60^Ni, ^63^Cu, ^66^Zn and ^111^Cd were compared with commercial standard curve preparations (Merck). ^107^Ag and ^115^In were used as internal standards.

### Evolutionary analysis by maximum likelihood method

The evolutionary history of the selected N‐carbamoyl‐β‐alanine amidohydrolase enzymes was inferred by using the maximum likelihood method and JTT matrix‐based model [53]. The tree with the highest log likelihood (−9451.94) is depicted in Fig. S1B. Initial tree(s) for the heuristic search were obtained automatically by applying neighbour‐join and BioNJ algorithms to a matrix of pairwise distances estimated using the JTT model and then selecting the topology with superior log likelihood value. The tree is drawn to scale, with branch lengths measured in the number of substitutions per site (next to the branches). This analysis involved 13 amino acid sequences. There were a total of 469 positions in the final dataset. Evolutionary analyses were conducted in mega x [54].

## Conflict of interest statement

The authors declare that they have no conflicts of interest with regard to this manuscript.

## Author contributions

AP, MK, SK and GA contributed to study conceptualisation. MK and AK contributed to investigation, initial microbial strain identification and characterisation; AP contributed to molecular biology; AP, AS, MK, CP and WAS contributed to protein purification and characterisation; AB and JM‐W contributed to structural biology; MDK, KD and HP contributed to preparation and validation of substrates. KYD contributed to metal analysis. JM‐W, GA, AP, AB and AH contributed to resources. AH, GA, AP and JM‐W contributed to funding acquisition. AP and JMW contributed to writing—original draft. AP, AH, JM‐W, AB, KD, AK and GA contributed to writing—review and editing. AP, AS, JM‐W and AB contributed to visualisation.

### Peer review

The peer review history for this article is available at https://www.webofscience.com/api/gateway/wos/peer‐review/10.1111/febs.16943.

## Supporting information


**Fig. S1.** Annotated sequence alignment and phylogenetic tree of carbamoylase enzymes.
**Fig. S2.** RrCβAA dimerisation interface.
**Fig. S3.** Rotation of the RrCβAA catalytic domain at the dimerisation domain boundary.
**Fig. S4.** Presence of electron density consistent with a MES buffer molecule in the RrCβAA active site.
**Fig. S5.** NMR spectra of ligands produced for this study.
**Fig. S6.** OPA derivatised amino acid standard curves for quantification of millimolar extinction coefficients.
**Fig. S7.** Bovine serum albumin standard curves for protein quantification by BCA.
**Table S1.** Purification table of recombinant RrCβAA.
**Table S2.** Temperature–activity relationship for RrCβAA.
**Table S3.** Temperature–stability relationship for RrCβAA.
**Table S4.** RrCβAA activity in the presence of divalent cations and reducing agents.
**Table S5.** Activity recovery of EDTA‐inactivated RrCβAA enzyme.
**Table S6.** Substrate preference of RrCβAA enzyme.

## Data Availability

All data used to prepare this manuscript are available as supplementary materials or deposited at publicly accessible databases. Links and references to datasets are in the Methods and Supplementary Materials.
