# Supplementary material for: Structural and biochemical characterisation of the N‐carbamoyl‐β‐alanine amidohydrolase from Rhizobium radiobacter MDC 8606
Source: FEBS J. 2023 Sep 8;290(23):5566–80. doi: 10.1111/febs.16943 (PMC10952681; doi:10.1111/febs.16943)
Supplement: Supplementary file 1 — Fig. S1. Annotated sequence alignment and phylogenetic tree of carbamoylase enzymes. Fig. S2. RrCβAA dimerisation interface. Fig. S3. Rotation of the RrCβAA catalytic domain at the dimerisation domain boundary. Fig. S4. Presence of electron density consistent with a MES buffer molecule in the RrCβAA active site. Fig. S5. NMR spectra of ligands produced for this study. Fig. S6. OPA derivatised amino acid standard curves for quantification of millimolar extinction coefficients. Fig. S7. Bovine serum albumin standard curves for protein quantification by BCA. Table S1. Purification table of recombinant RrCβAA. Table S2. Temperature–activity relationship for RrCβAA. Table S3. Temperature–stability relationship for RrCβAA. Table S4. RrCβAA activity in the presence of divalent cations and reducing agents. Table S5. Activity recovery of EDTA‐inactivated RrCβAA enzyme. Table S6. Substrate preference of RrCβAA enzyme. [file FEBS-290-5566-s001.docx]

### Supplementary Information

### Structural and biochemical characterisation of the N-carbamoyl-β-alanine Amidohydrolases from *Rhizobium radiobacter* MDC 8606

Ani Paloyan^1*^, Armen Sargsyan^1^, Mariam D. Karapetyan^1^, Artur Hambardzumyan^1^, Sergei Kocharov^2^, Henry Panosyan^2,^ Karine Dyukova^1^, Marina Kinosyan^1^, Anna Krueger^3^, Cecilia Piergentili^4^, Will A. Stanley^4^, Karrera Y Djoko^5^, Arnaud Baslé^6^, Jon Marles-Wright^4,6*^, Garabed Antranikian^7^

^1^Scientific and Production Center “Armbiotechnology” of NAS RA, 14 Gyurjyan Str., 0056 Yerevan, Armenia

^2^The Scientific Technological Centre of Organic and Pharmaceutical Chemistry SNPO of NAS RA, 26 Azatutyan ave., 0014 Yerevan, Armenia

^3^Authority for the Environment, Climate, Energy and Agriculture in Hamburg, 21109 Hamburg, Germany

^4^School of Natural and Environmental Sciences, Newcastle University. NE1 7RU Newcastle upon Tyne, UK

^5^Department of Biosciences, Durham University, Durham, UK

^6^Newcastle University Biosciences Institute, Faculty of Medical Sciences, Newcastle University, Newcastle upon Tyne, UK

^7^Center for Biobased Solutions TUHH, 21073 Hamburg, Germany

* To whom correspondence should be addressed

Jon Marles-Wright, +44(0)191 208 4855, [Jon.marles-wright1@ncl.ac.uk](about:blank);

Ani Paloyan, +374 94934664, [ani_paloyan@ysu.am](mailto:ani_paloyan@ysu.am)

**Contents**

**Supplementary Figures**

Supplementary Figure 1. Annotated sequence alignment and phylogenetic tree of carbamoylase enzymes

Supplementary Figure 2. RrCβAA dimerization interface

Supplementary Figure 3. Rotation of the RrCβAA catalytic domain at the dimerization domain boundary

Supplementary Figure 4. Presence of electron density consistent with a MES buffer molecule in the RrCβAA active site

Supplementary Figure 5. NMR spectra of ligands produced for this study

Supplementary Figure 6. OPA derivatised amino acid standard curves for quantification of millimolar extinction coefficients

Supplementary Figure 7. Bovine serum albumin standard curves for protein quantification by BCA.

**Supplementary Tables**

Supplementary Table 1. Purification table of recombinant RrCβAA

Supplementary Table 2. Temperature - activity relationship for RrCβAA

Supplementary Table 3. Temperature - stability relationship for RrCβAA

Supplementary Table 4. RrCβAA activity in the presence of divalent cations and reducing agents

Supplementary Table 5. Activity recovery of EDTA inactivated RrCβAA enzyme

Supplementary Table 6. Substrate preference of RrCβAA enzyme


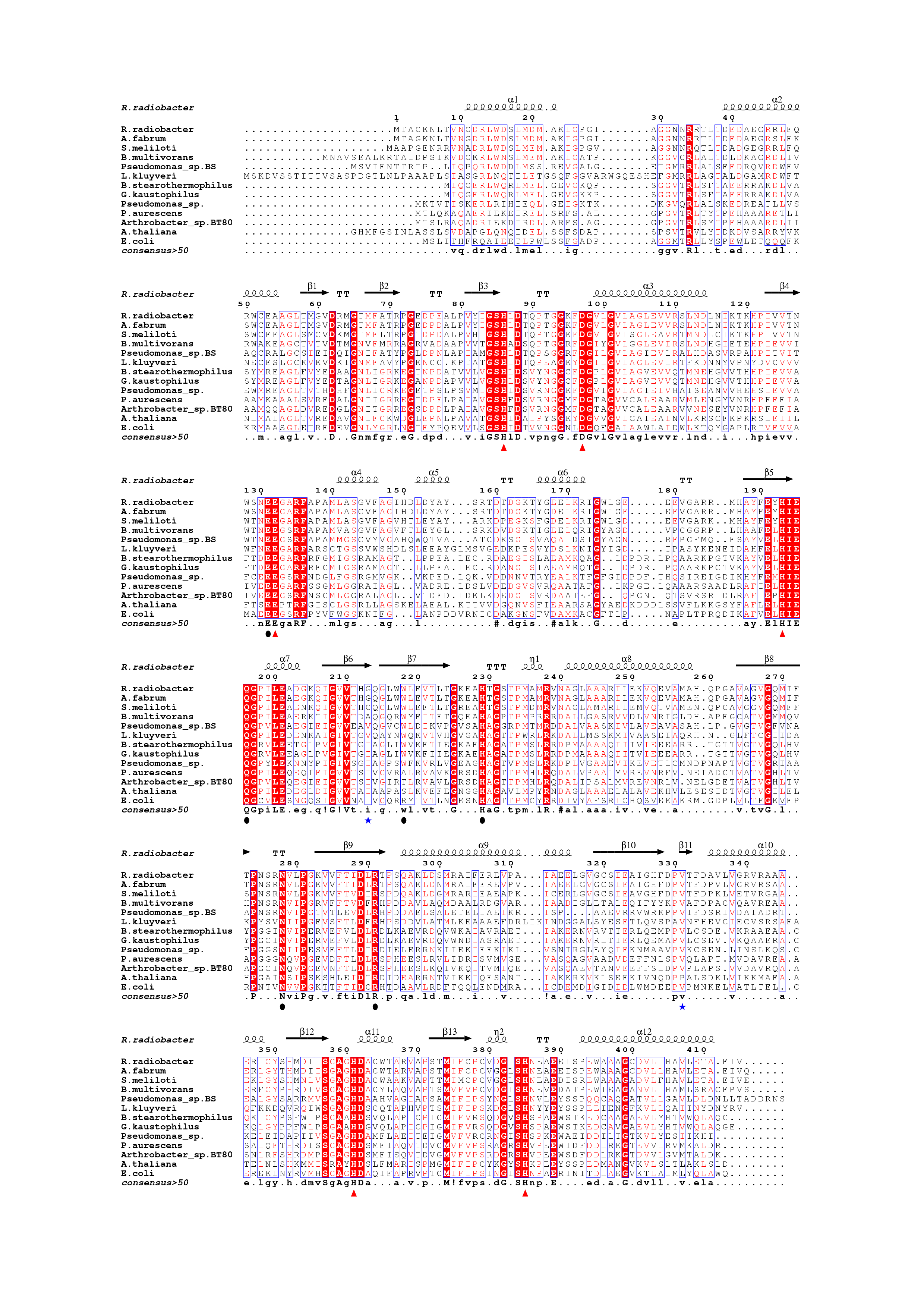


**
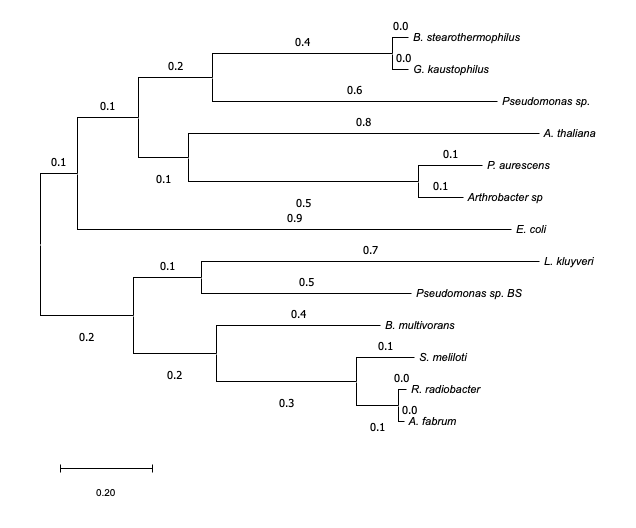
**

**Supplementary Figure 1. Annotated sequence alignment and phylogenetic tree of carbamoylase enzymes.**

A) multiple sequence alignment of carbamoylase enzymes was performed using Multalin and visualised with ESPript. Secondary structure elements from the crystal structure of the *R. radiobacter* enzyme are shown above the alignment. Strictly conserved residues are shown with a red background, while partially conserved residues are shown with red text. Conserved residues involved in metal coordination are highlighted with red triangles, residues involved in ligand binding with black ovals and hinge residues between the active site and dimerisation domains are shown with blue stars. B) Phylogenetic tree depicting the evolutionary relationship of the carbamoylase enzymes shown in panel A. Tree prepared using MegaX according to program settings as outlined in materials and methods section.

**
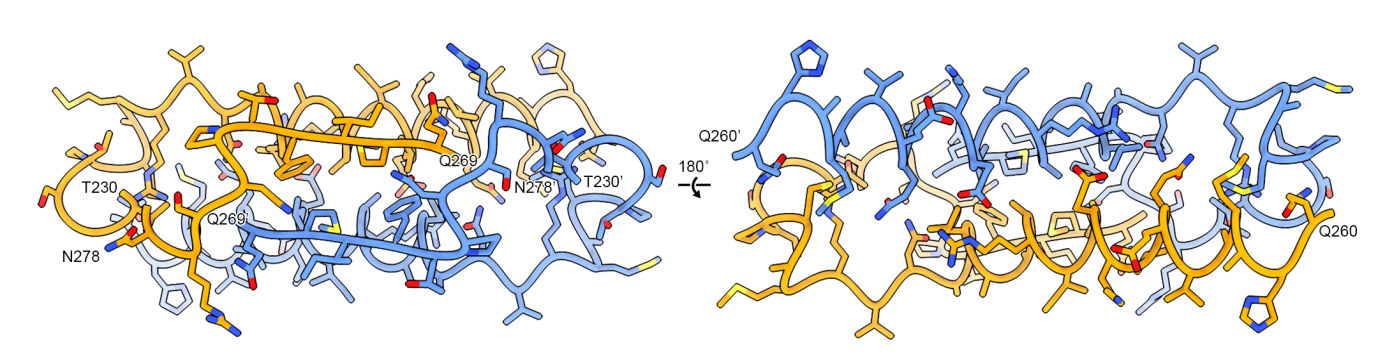
**

**Supplementary Figure 2. RrCβAA dimerization interface.** The RrCβAA dimerization interface is shown as cartoon backbone representation with interacting residues shown as sticks coloured by atom. The subunits are coloured orange and blue, with residues from the blue subunit indicated with a prime symbol. Figure prepared using ChimeraX version 1.6.1.

**
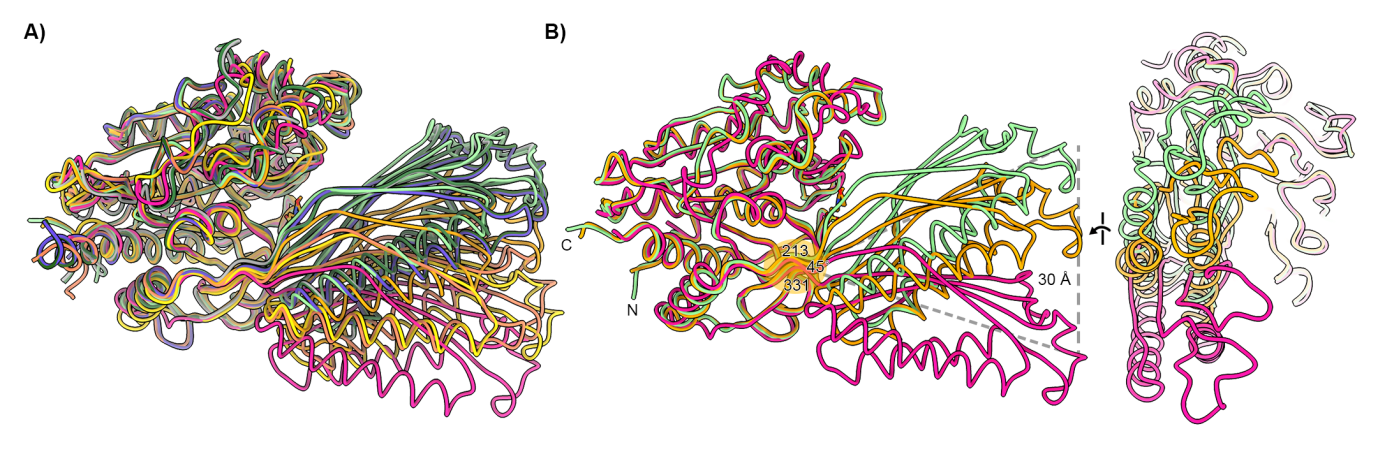
Supplementary Figure 3. Rotation of the RrCβAA catalytic domain at the dimerization domain boundary.** A) Range of rotation present in published homologue structures, shown as licorice representation. Models are coloured as follows: 8APZ – mint; 5THW – peach; 3N5F – pink;1R3N – salmon; 1Z2L – yellow; 2V8G – purple; 4PXB – grey; RrCβAA – Orange. B) Extremes of rotation between the domains highlighted with 8APZ and 3N5F – the hinge residues in RrCβAA are highlighted with the maximum angle of rotation and extent of relative displacement of the catalytic domain to the dimerization domain. Figure prepared using ChimeraX version 1.6.1.

**
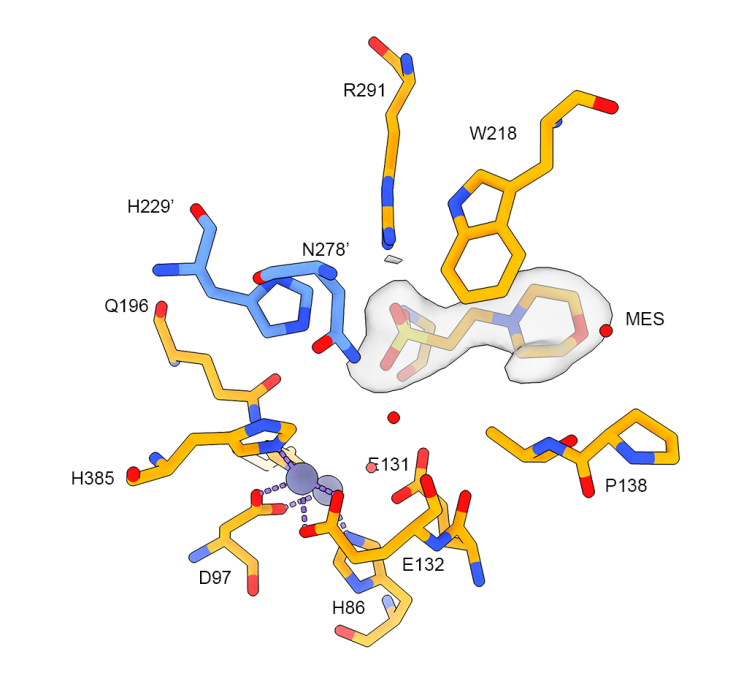
Supplementary Figure 4. Presence of electron density consistent with a MES buffer molecule in the RrCβAA active site.** Based on features in the 2mFo-DFc electron density map a MES buffer molecule was modelled in the active site. Residues interacting with the MES molecule are shown as stick representation in orange and blue, with the carbon atoms of the MES molecule shown in orange. Final 2mFo-DFc Electron density map rendered at 1σ as a transparent grey surface. Figure prepared using ChimeraX version 1.6.1.

**Supplementary Figure 5. NMR spectra of ligands produced for this study.**

**Compound 1: 4-(Methylthio)-2-ureidobutanoic acid.** From L-methionine. M. p. 182-183 ^o^C (EtOH-H_2_O, 5:1). ^1^H NMR δ: 1.74 – 1.86 (m, 1H) and 1.92 – 2.03 (m, 1H, both CH_2_); 2.06 (s, 3H, CH_3_); 2.47 (t, 2H, J = 7.7 Hz, SCH_2_); 4.19 (td, 1H, J = 8.2, 4.9 Hz, CH); 5.50 (br., 2H, NH_2_); 6.30 (d, 1H, J = 8.2 Hz, NH); 12.52 (br., 1H, COOH). ^13^C NMR δ: 14.7 (CH_3_); 29.6 (CH_2_); 31.9 (CH_2_); 51,4 (CH); 158.4 (NCO); 174.1 (OCO).
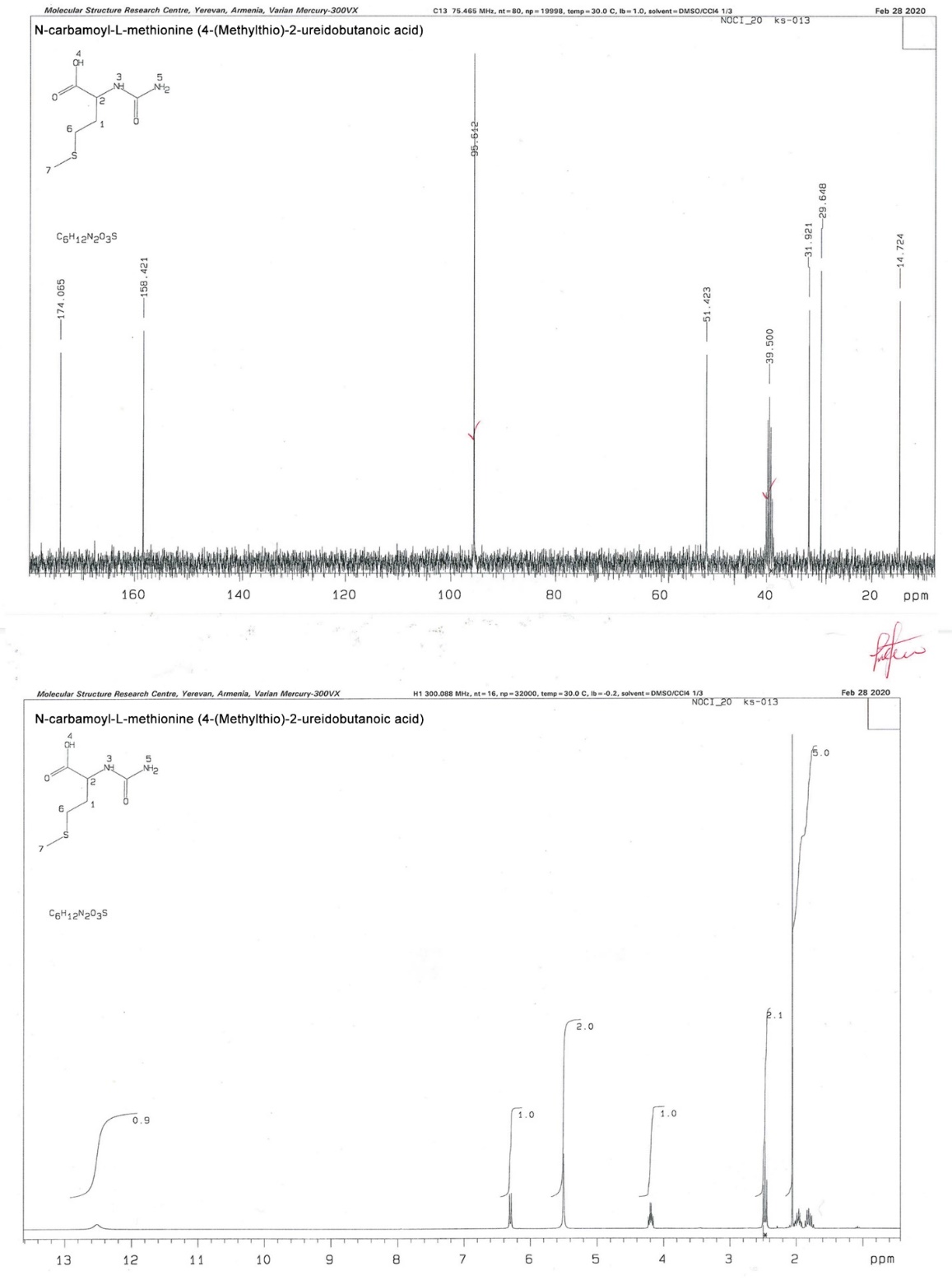


**Compound 2: 4-Methyl-2-ureidopentanoic acid.** From L-leucine.M. p. 227-230 ^o^C (H_2_O) (lit.: 216-218 ^o^C) [1]. ^1^H NMR δ: 0.92 (d, 3H, J = 6.5 Hz) and 0.93 (d, 3H, J = 6.5 Hz, both 2CH_3_); 1.37 – 1.55 (m, 2H, CH_2_); 1.63 – 1.80 (m, 1H, CH_3_C**H**CH_3_); 4.11 (ddd, 1H, J = 8.9, 8.6, 5.3 Hz, CH); 5.40 (br., 2H, NH_2_); 6.12 (d, 1H, J = 8.6 Hz, NH); 12.25 (v.br., 1H, COOH). ^13^C NMR δ: 21.6 (CH_3_); 22.7 (CH_3_); 24.2 (CH); 41.3 (CH_2_); 50.6 (NCH); 158.3 (NCO); 175,1 (OCO).


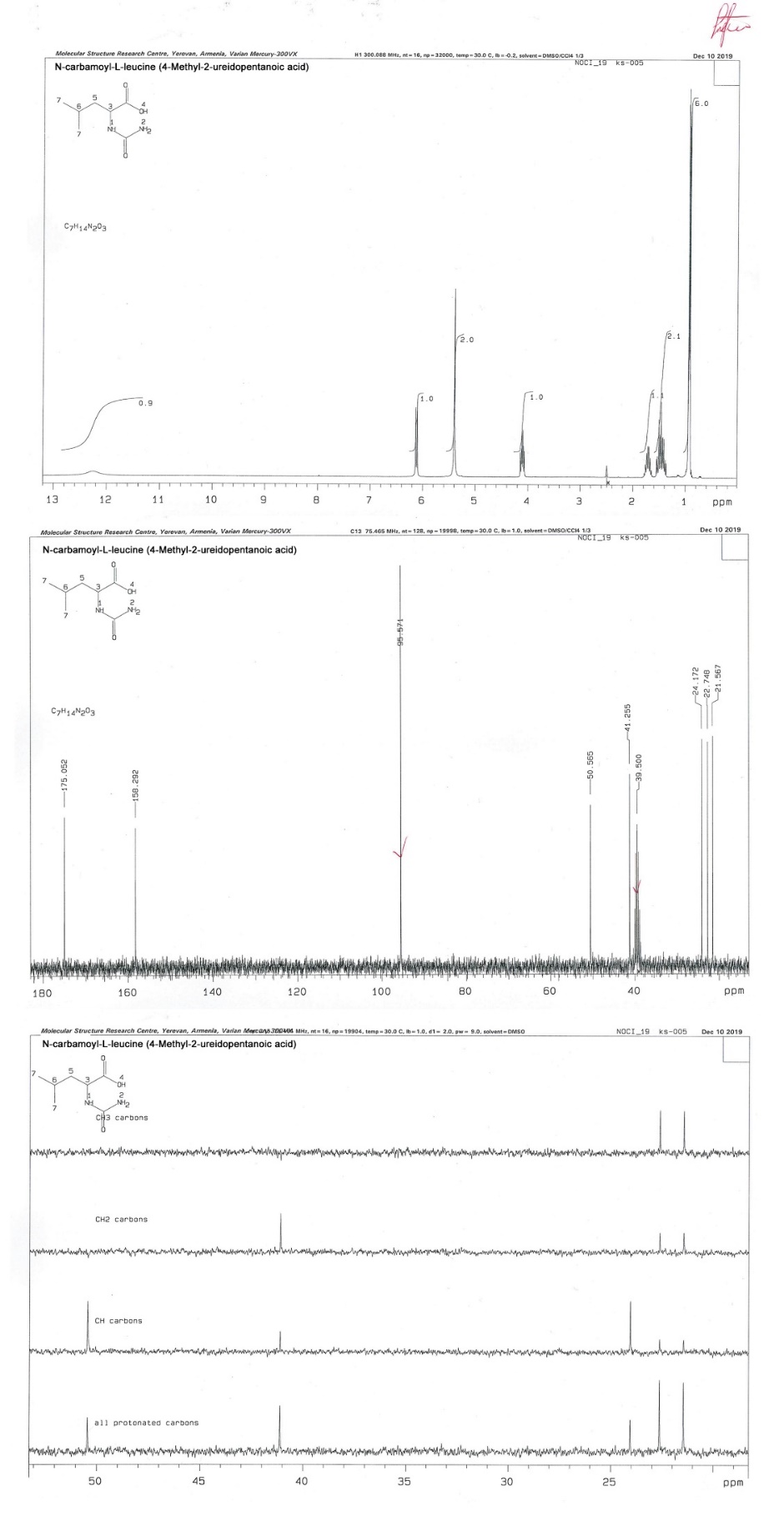


**Compound 3: 4-Ureidobutanoic acid**. From 4-aminobutanoic acid. M. p. 187-188 ^o^C (EtOH-H_2_O, 5:1), lit.: 174-175 ^o^C [ 2 ], 178-179 ^o^C [ 3 ]. ^1^H NMR δ: 1.65 (tt, 2H, J = 7.4, 6.7 Hz, CH_2_); 2.21 (t, 2H, J = 7.4 Hz, O=C-CH_2_); 3.02 (td, 2H, J = 6.7, 5.8 Hz, NCH_2_); 5.21 (br., 2H, NH_2_); 5.91 (br. t, 1H, J = 5.8 Hz, NH); 11.88 (br., 1H, COOH). ^13^C NMR δ: 25.4 (CH_2_); 30,9 (CH_2_); 38.3 (CH_2_); 158.6 (NCO); 173.9 (OCO).


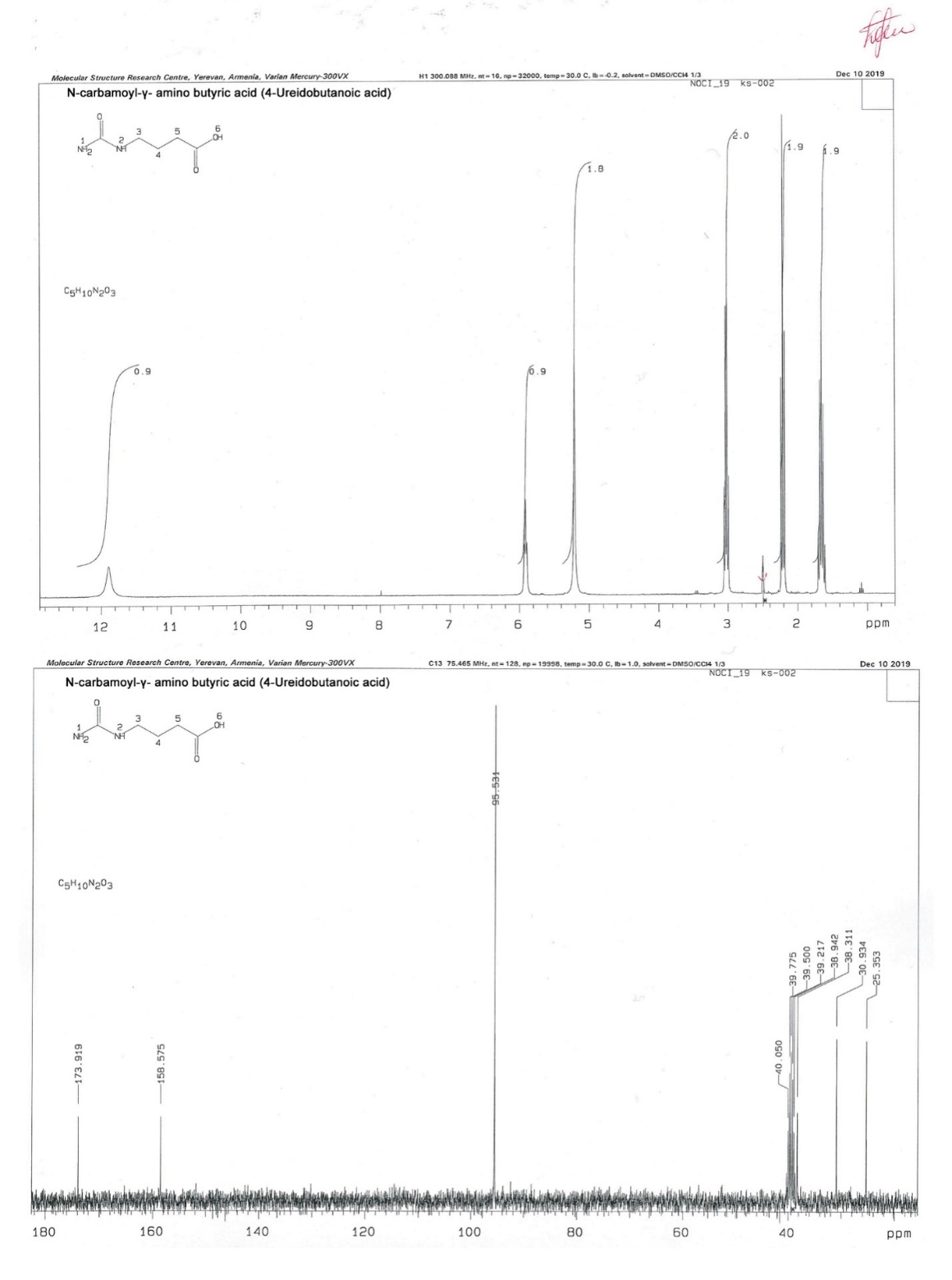


**Compound 4: 2-Ureidobutanoic acid.** From DL-2-aminobutanoic acid. M. p. 196-197 ^o^C (EtOH-H_2_O, 5:1), lit.: 178-179 ^o^C [ 3 ]. ^1^HNMR δ: 0.91 (t, 3H, J = 7.4 Hz, CH_3_); 1.53 – 1.80 (m, 2H, CH_2_); 4.06 (ddd, 1H, J = 7.83, 7.1, 5.3 Hz, CH); 5.50 (br., 2H, NH_2_); 6.22 (d, 1H, J = 8.3 Hz, NH); 12.40 (br., 1H, COOH). ^13^C NMR δ: 9.6 (CH_3_); 25.3 (CH_2_); 53.3 (CH); 158.5 (NCO); 174.4 (OCO).


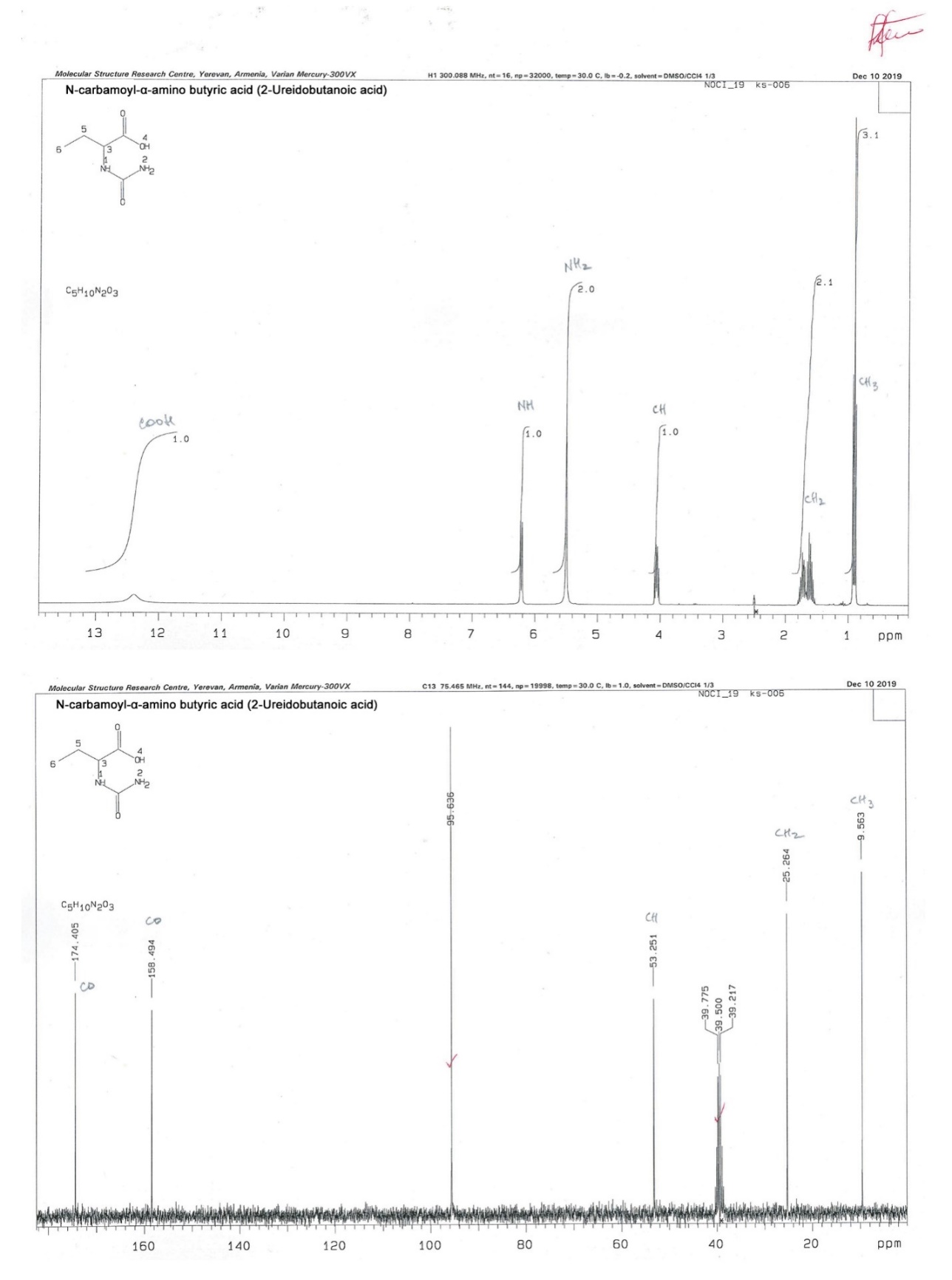


**Compound 5: 3-Phenyl-2-ureidopropionic acid.** From DL-phenylalanine. M. p. 198 ^o^C (H_2_O). ^1^H NMR δ: 2.93 (dd, 1H, J = 13.7, 7.0 Hz) and 3.04 (dd, 1H, J = 13.7, 5.4 Hz, both CH_2_); 4.41 (ddd, 1H, J = 8.2, 7.0, 5.4 Hz, CH); 5.46 (br., 2H, NH_2_); 6.14 (d, 1H, J = 8.2 Hz, NH); 7.13 – 7.28 (m, 5H, Ar); 12.41 (v.br., 1H, COOH).^13^C NMR δ: 37.2 (CH_2_); 52.9 (CH); 125.3 (CH); 127.1 (2CH); 128.6 (2CH); 136.7; 157.4 (NCO); 173.0 (OCO).


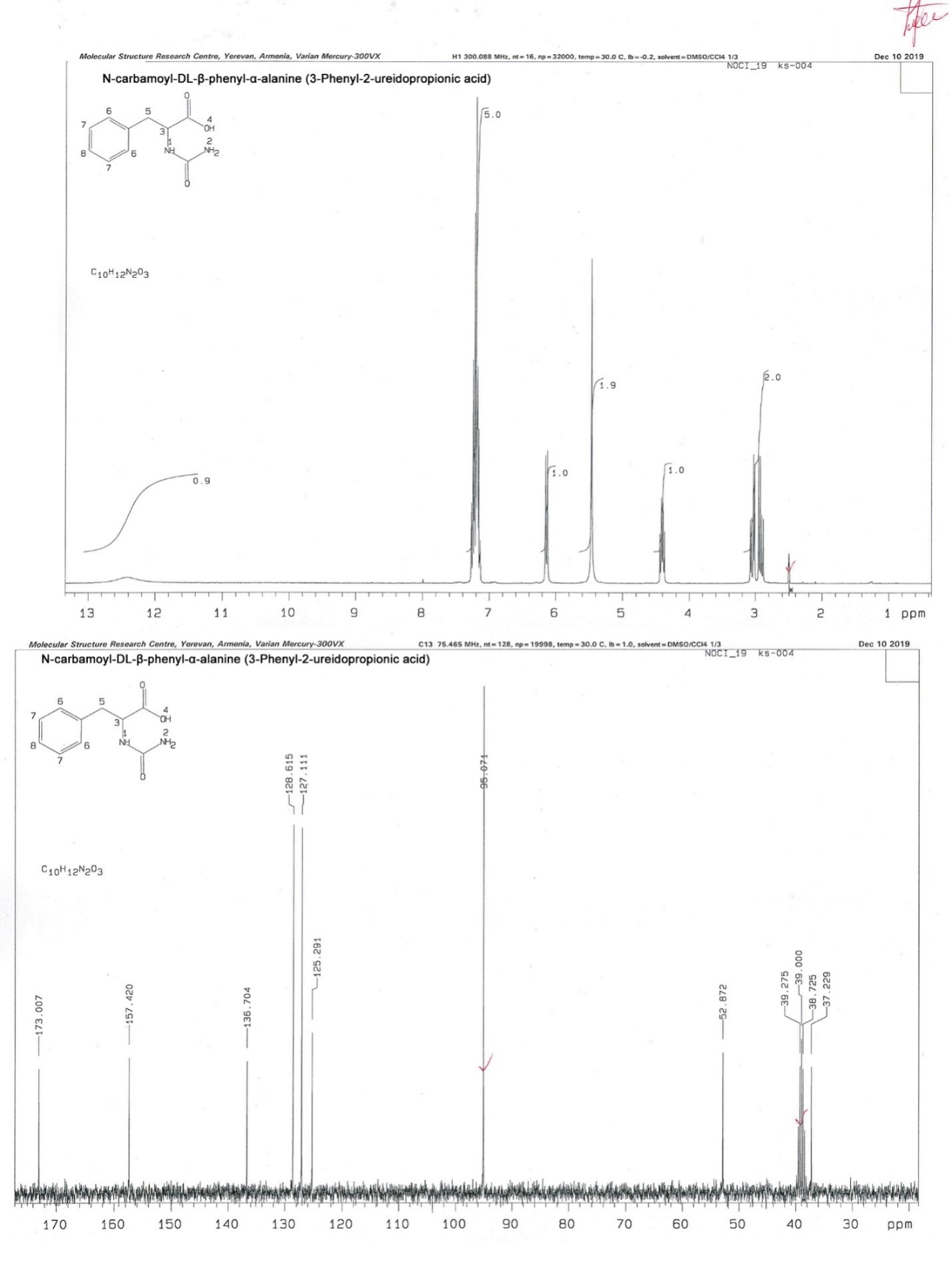


**Compound 6: 3-Phenyl-2-ureidopropanoic acid.** From L-phenylalanine. M. p. 210-211 ^o^C (H_2_O) (lit.: 200 ^o^C) [1]. Data of ^1^H and ^13^C NMR are same as for **5**.


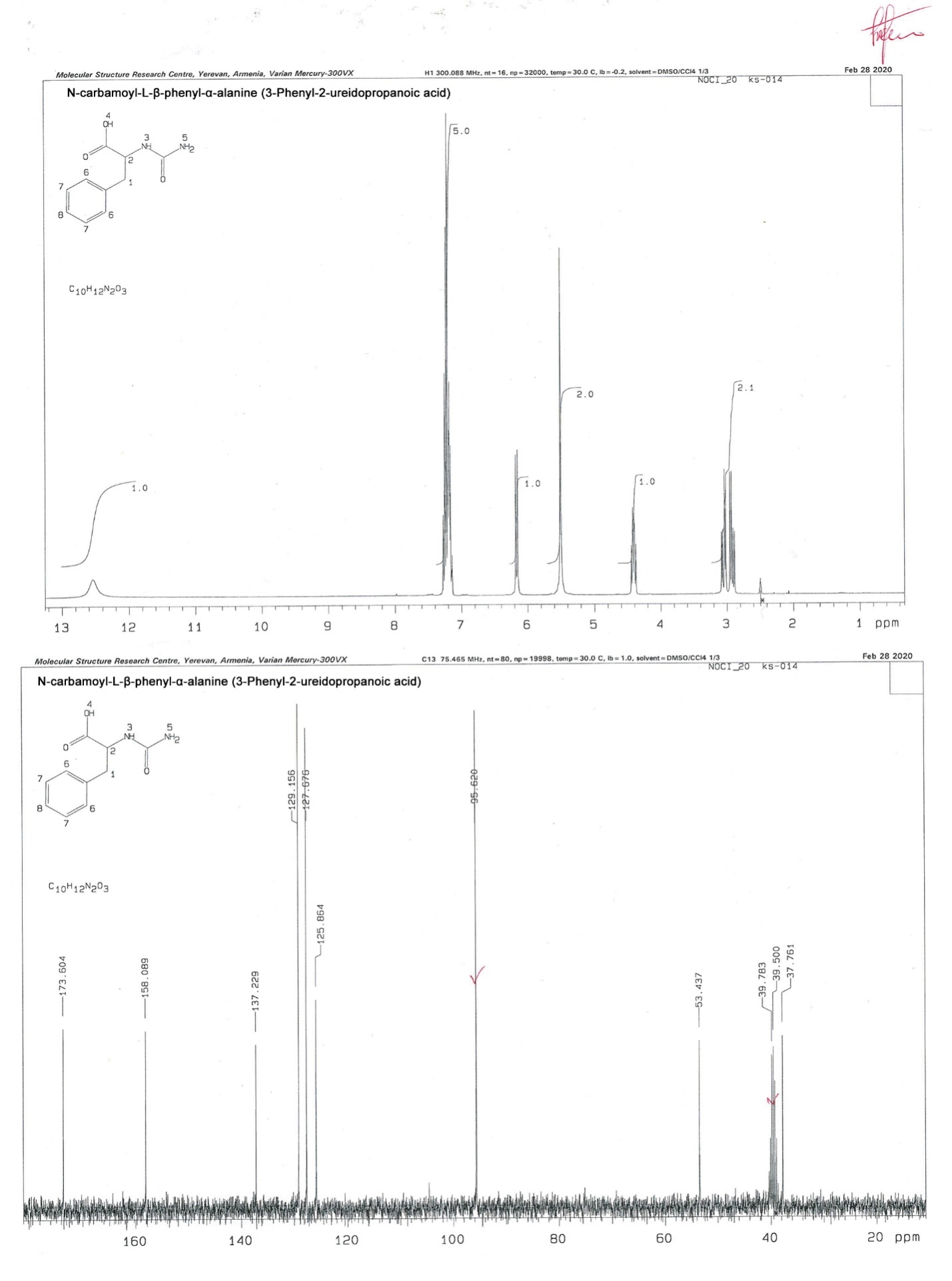


**Compound 7: 3-Phenyl-3-ureidopropanoic acid.** From DL-3-amino-3phenylpropanoic acid. M. p. 203-206 ^o^C (EtOH). ^1^H NMR δ: 2.62 (dd, 1H, J = 15.3, 6.3 Hz) and 2.66 (dd, 1H, J = 15.3, 7.6 Hz, both CH_2_); 5.05 (ddd, 1H, J = 8.6, 7.6, 6.3 Hz, CH); 5.39 (br. 2H, NH_2_); 6.55 (d, 1H, J = 8.6 Hz, NH); 7.15-7.33 (m, 5H, Ar); 12.10 (br. 1H, COOH). ^13^C NMR δ: 41.4 (CH_2_); 49.9 (CH); 126.0 (2CH); 126.2 (CH); 127.7 (2CH); 143.2; 157.8 (NCO); 171.8 (OCO).


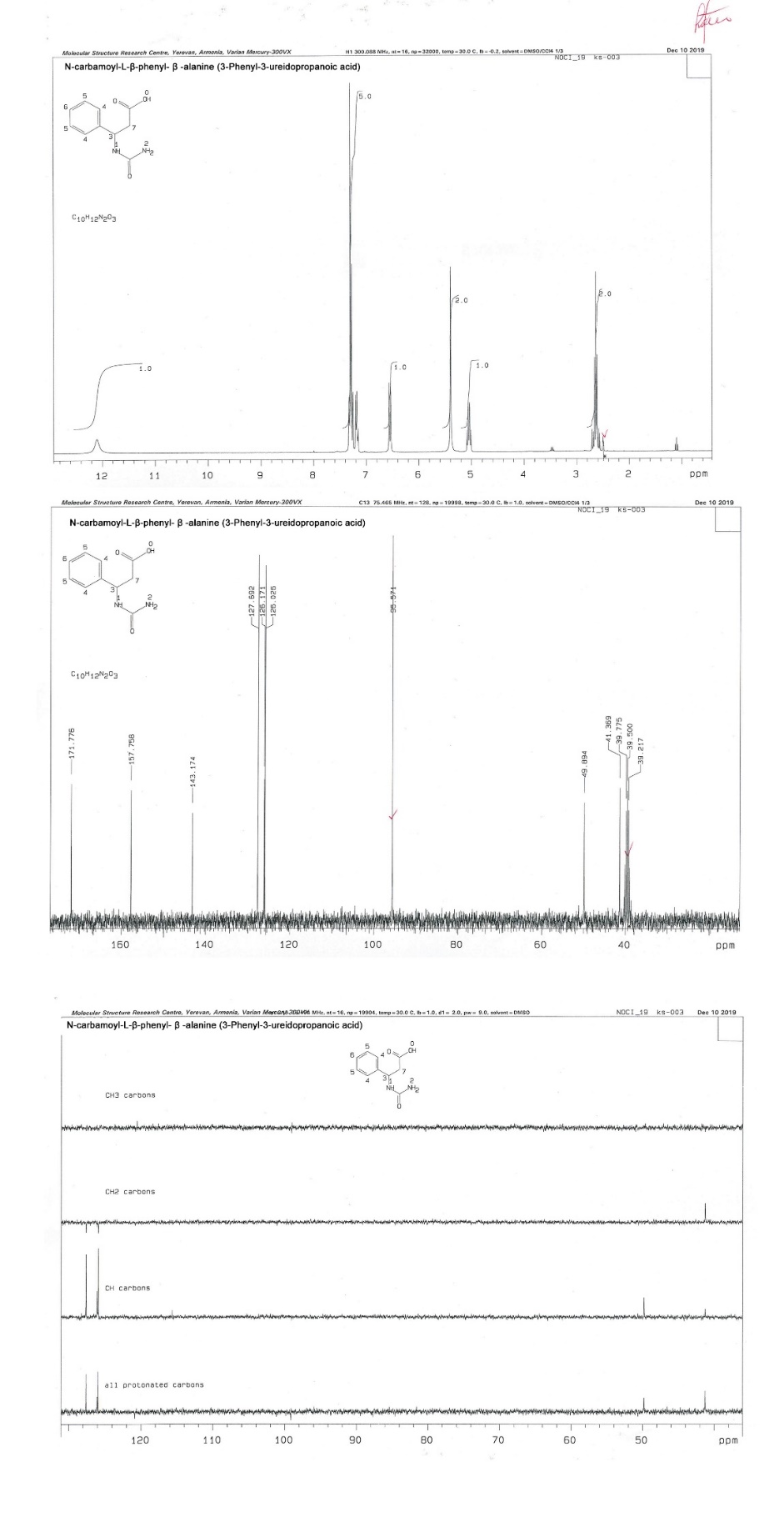


**Compound 8: 3-Methyl-2-ureidobutanoic acid.** From DL-valine. M. p. 211-213 ^o^C (EtOH-H_2_O, 5:1). M. p. 218-220 ^o^C (EtOH-H_2_O, 5:1), lit.: 207-209 ^o^C [1]. ^1^H NMR δ: 0.88 (d, 3H, J = 6.8 Hz) and 0.93 (d, 3H, J = 6.8 Hz, both 2CH_3_); 2.05 (dsp, 1H, J = 6.8, 4.9 Hz, CH_3_C**H**CH_3_); 4.05 (dd, 1H, J = 9.1, 4.9 Hz, NCH); 5.43 (br., 2H, NH_2_); 6.13 (br. d, 1H, J = 9.1 Hz, NH); 12.28 (br., 1H, COOH). ^13^C NMR δ: 17.4 (CH_3_); 18.9 (CH_3_); 30.3 (CH); 57.0 (NCH); 158.4 (NCO); 173.9 (OCO).


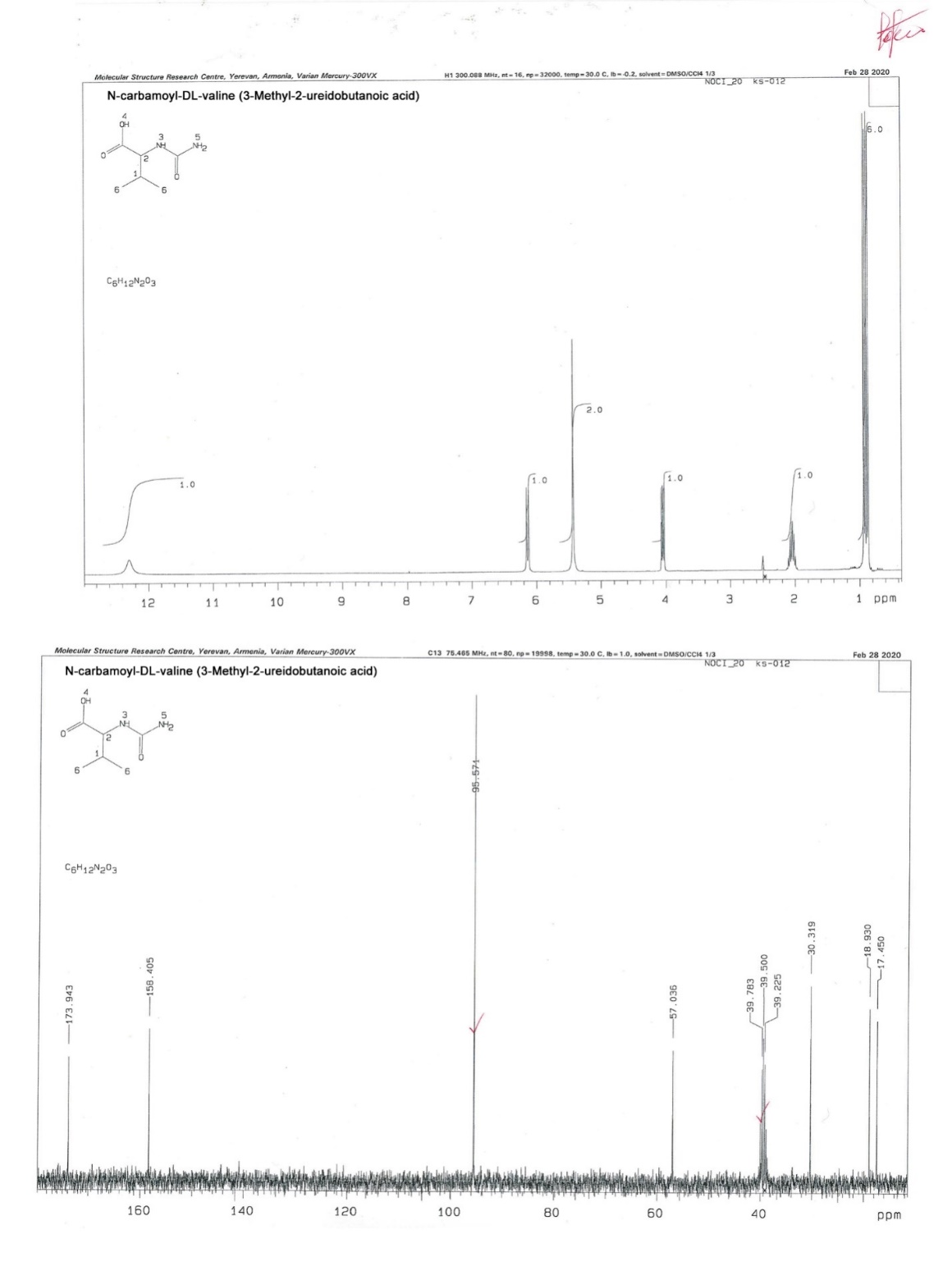


**Compound 9: 3-Methyl-2-ureidobutanoic acid.** From D-valine. M. p. 225-226 ^o^C (EtOH-H_2_O, 5:1). Data of ^1^H and ^13^C NMR are same as for **8**.


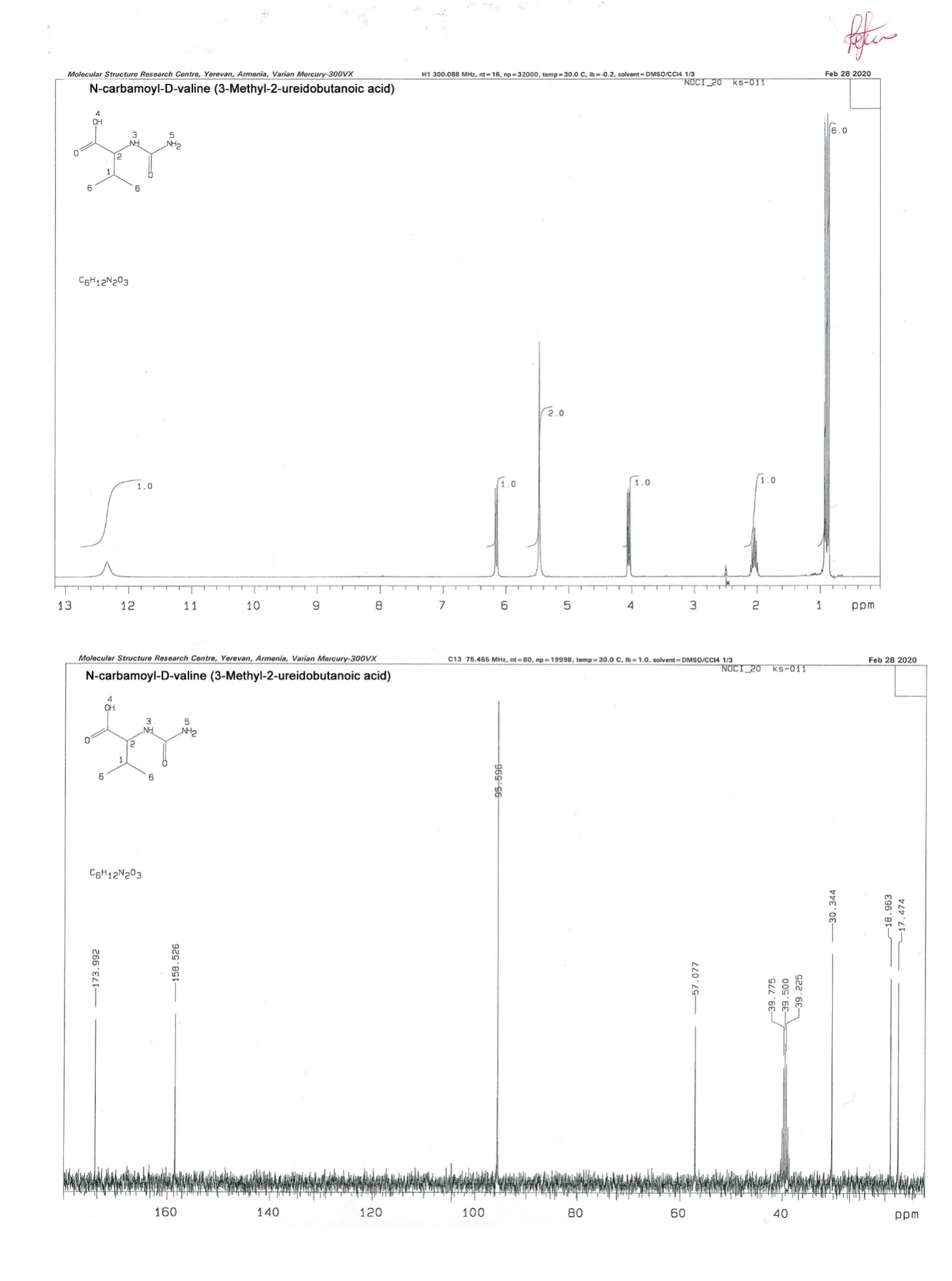


**Compound 10: 3-Methyl-2-ureidobutanoic acid.** From L-valine. M. p. 218-220 ^o^C (EtOH-H_2_O, 5:1), lit.: 207-209 ^o^C [1]. Data of ^1^H and ^13^C NMR are same as for **8.**


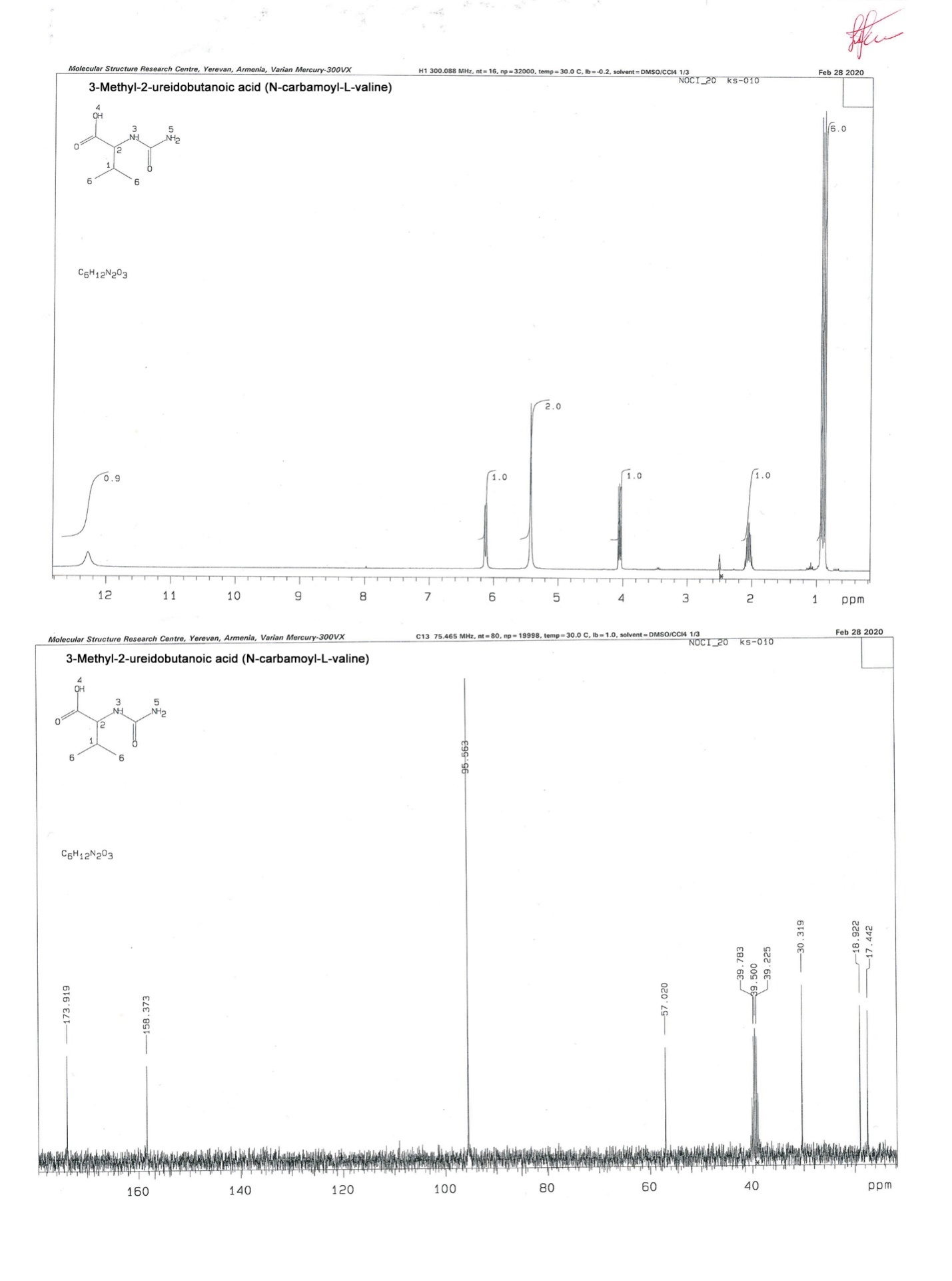


**Compound 11: 2-Ureidopropanoic acid.** From DL-Alanine. M. p. 187-189 ^o^C (EtOH-H_2_O, 2:1), lit.: 185 ^o^C [4]. ^1^H NMR δ: 1.28 (d, 3H, J = 7.3 Hz, CH_3_); 4.11 (dq, 1H, J = 7.8, 7.2 Hz, CH); 5.41 (br. 2H, NH_2_); 6.18 (br. d, 1H, J = 7.8 Hz, NH); 12.31 (br., 1H, COOH). ^13^C NMR δ: 18.3 (CH_3_); 47.7 (CH); 158.0 (NCO); 174.9 (OCO).


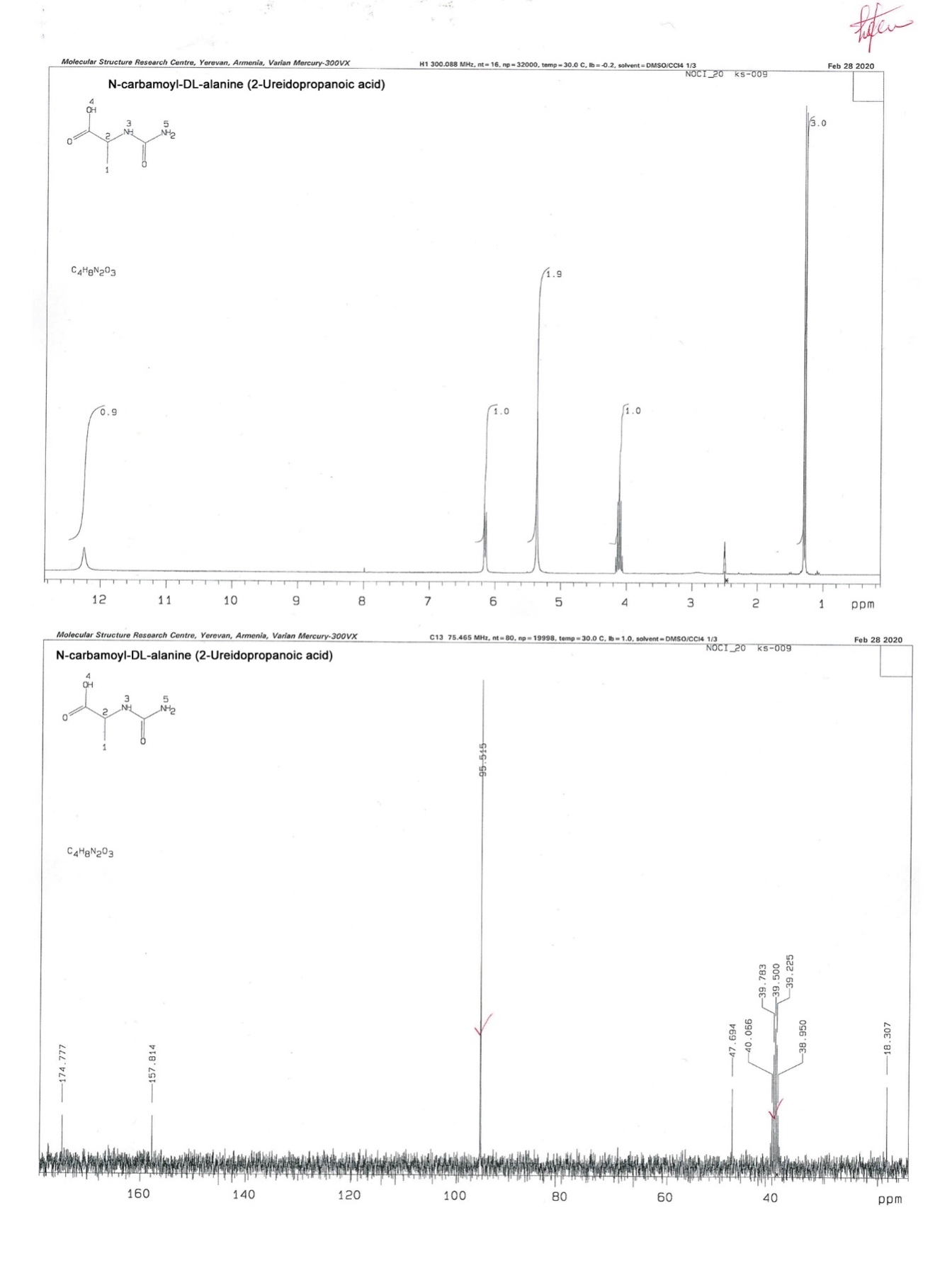


**Compound 12: 2-Ureidopropanoic acid.** From D-alanine. M. p. 201.5-202 ^o^C (EtOH-H_2_O, 2:1). Data of ^1^H and ^13^C NMR are same as for **11**.


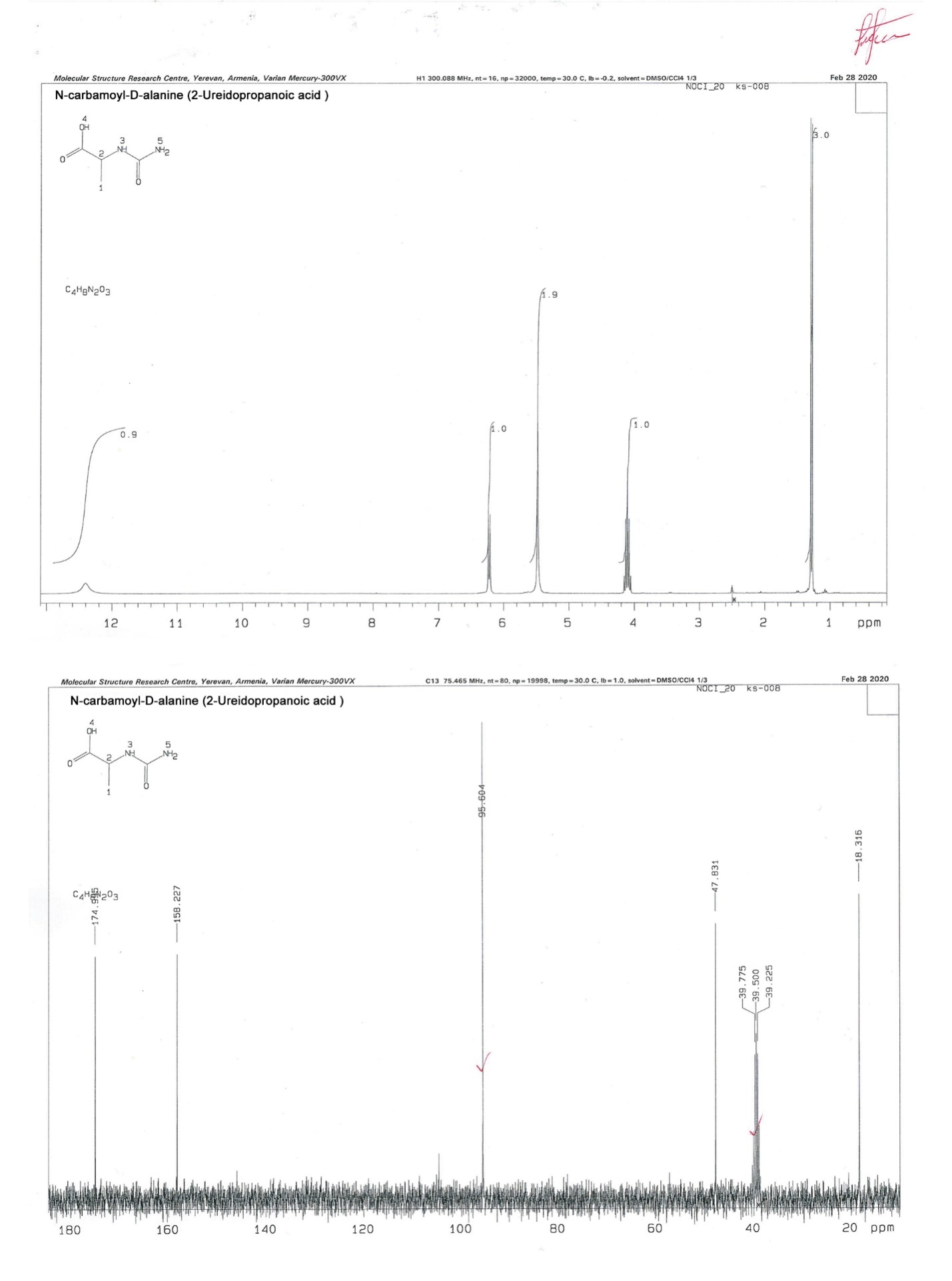


**Compound 13: 2-Ureidopropanoic acid.** From L-alanine. M. p. 208-209 ^o^C (EtOH-H_2_O, 2:1), lit.: 165-167 ^o^C [1], 198-200 ^o^C[4 ].Data of ^1^H and ^13^C-NMR are same as for **11**.


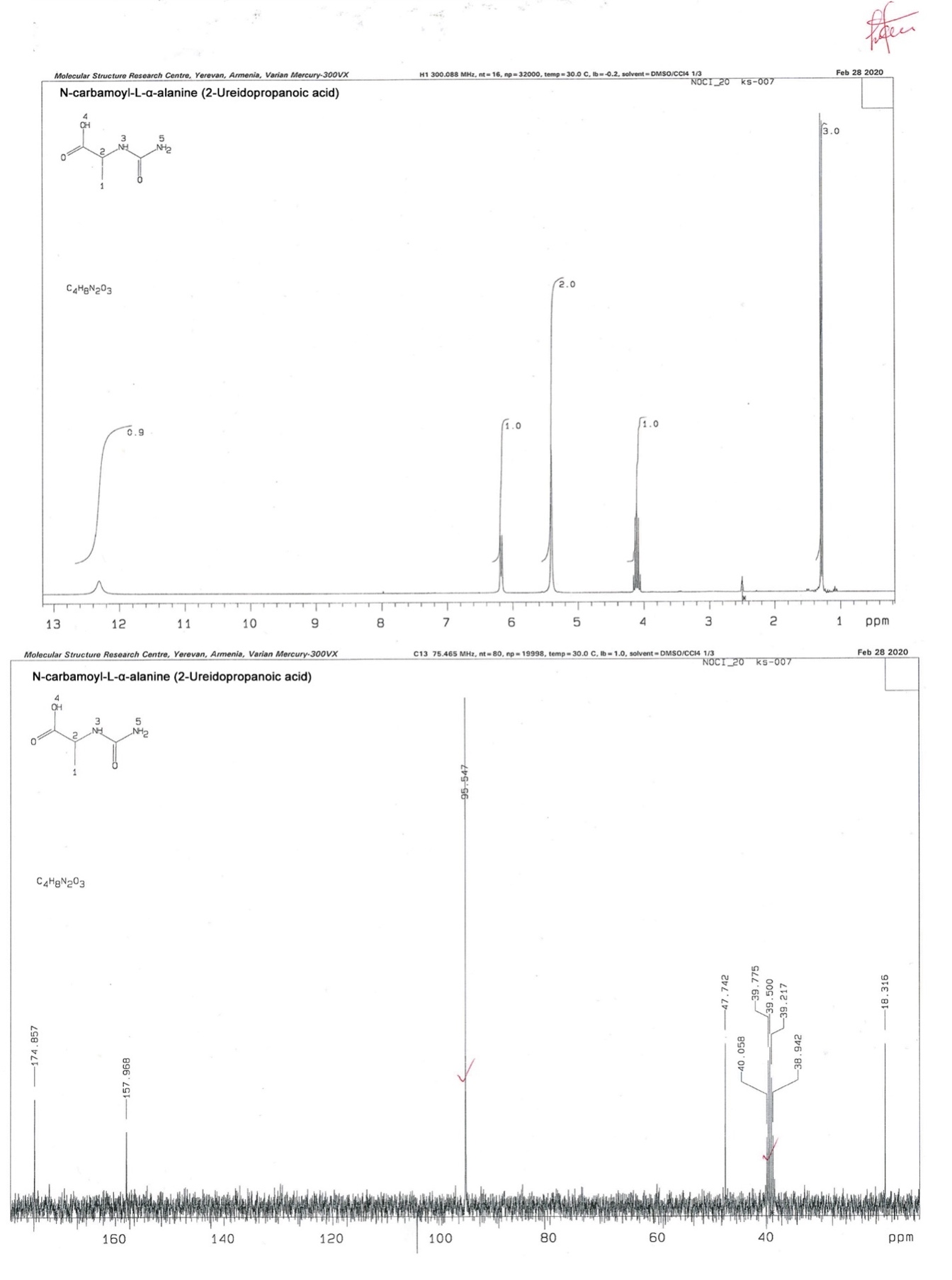


**Compound 14: N-Carbamoyl-3-(4-isopropoxyphenyl)-4-aminobutanoic acid.** From previously synthesized 3- (4-isopropoxyphenyl)-4-aminobutanoic acid [5].M.p. 217-219 ^o^C (EtOH).^1^H NMR δ: 1.30 (d, 6H, J = 6.0 Hz, 2CH_3_); 2.38 (dd, 1H, J = 15.6, 8.4 Hz, CH_2_); 2.59 (dd, 1H, J = 15.6, 5.7 Hz, CH_2_); 3.04 – 3.28 (m, 3H, NCH_2_CH); 4.51 (sp, 1H, J= 6.0 Hz, OCH); 5.21 (br., 2H, NH_2_); 5.79 (br.t, 1H, J = 5.5 Hz, NH); 6.74 – 6.79 (m, 2H, Ar); 7.08 – 7.13 (m, 2H, Ar); 11,79 v.br.(1H, COOH). ^13^C NMR δ: 21.7 (2CH_3_); 37,9 (CH_2_); 41,3 (CH); 44,5 (NCH_2_); 68.7 (OCH); 115.0 (2CH); 128.2 (2CH); 134.0; 155.8; 158.4; 172,9.


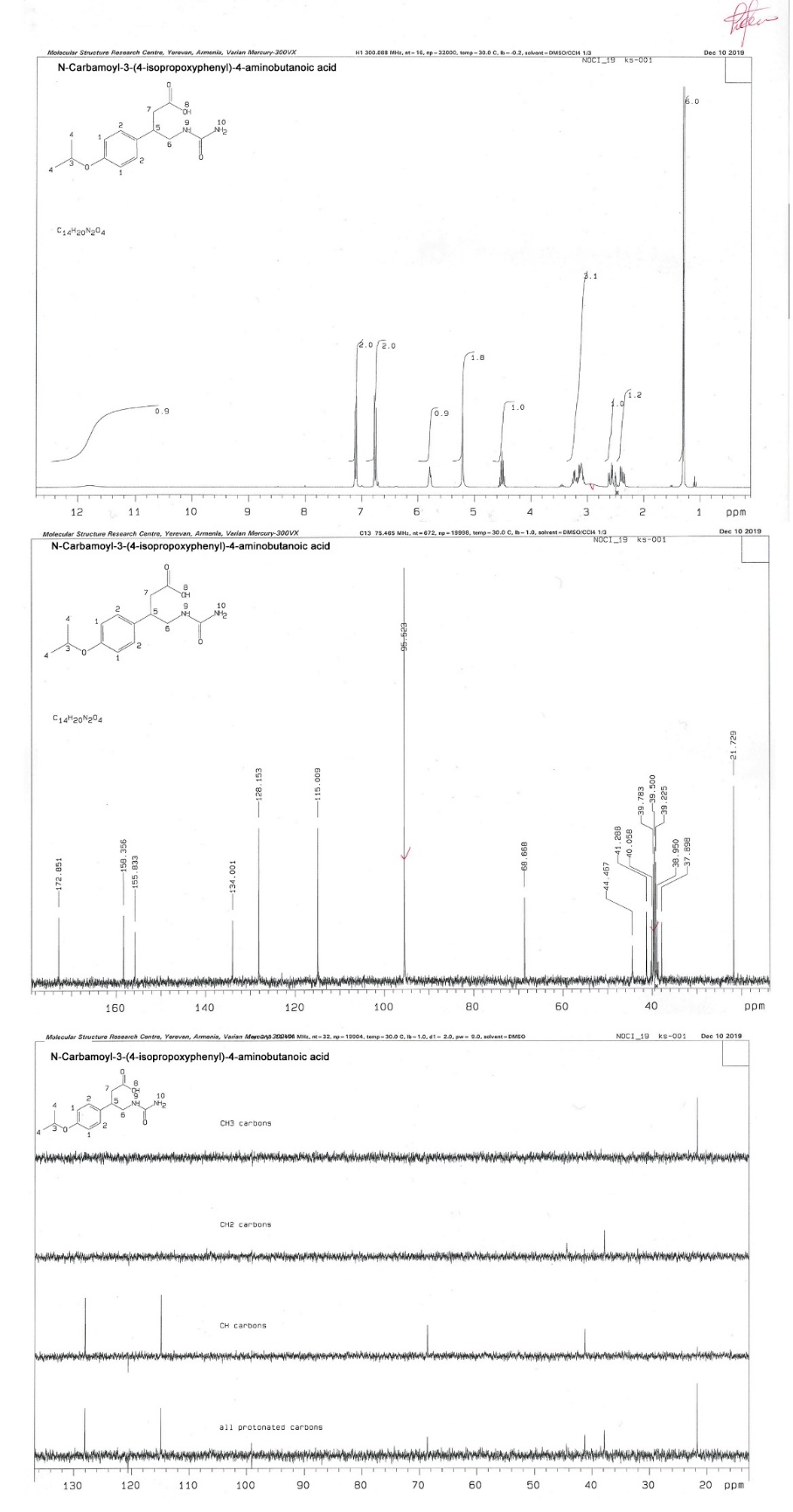


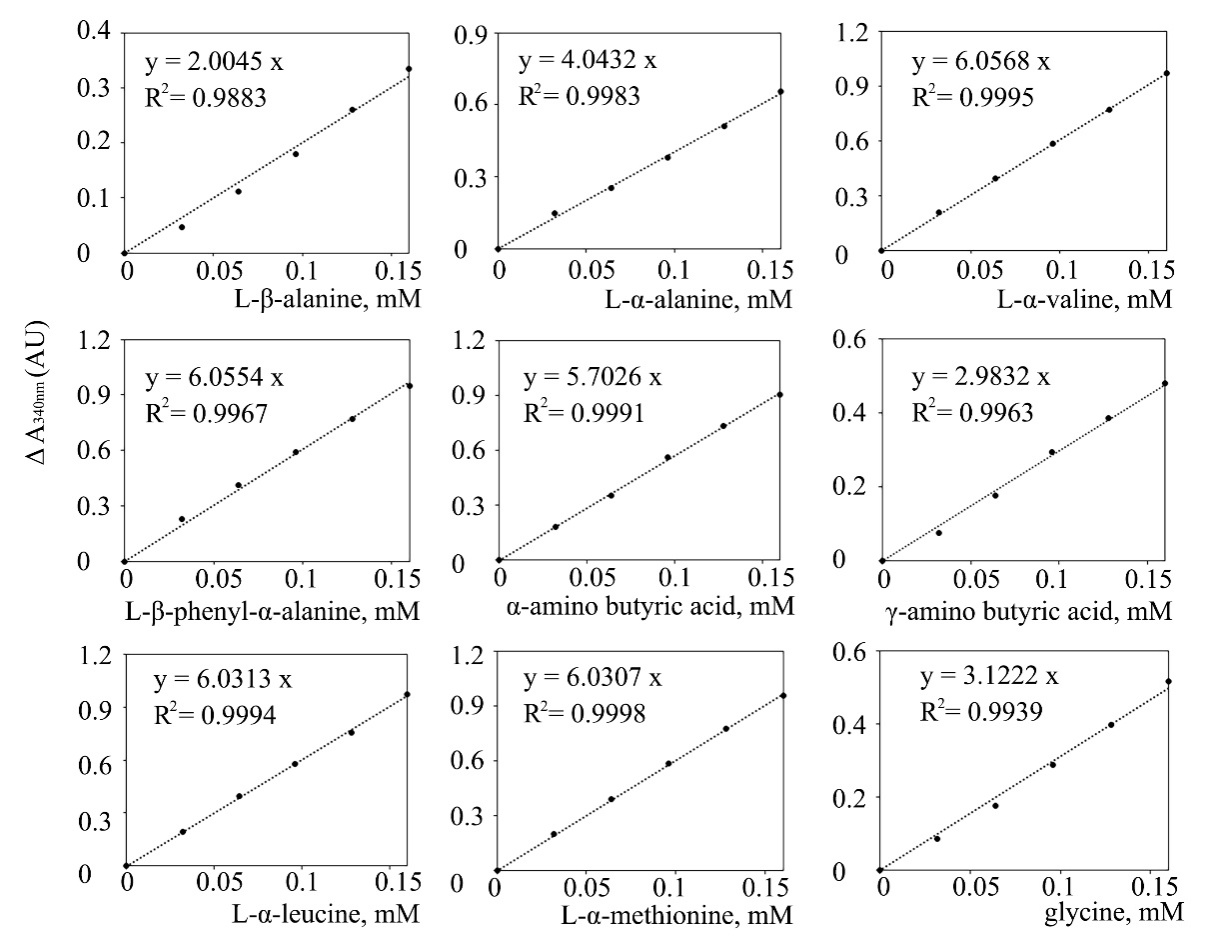


**Supplementary Figure 6. OPA derivatised amino acid standard curves for quantification of millimolar extinction coefficients.** All standard curves were generated by using a plot of absorbance vs. concentration at 340 nm to determine the molar extinction coefficient of amino acids. The samples were prepared at concentrations of 0, 0.032, 0.064, 0.096, 0.128 and 0.16 mM with 3 mL of freshly prepared activity reagent (0.1 M sodium borate pH 9.2, 2 mM OPA and 5 mM β-mercaptoethanol). After incubation at 20 °C for 30 min the absorption of the corresponding isoindole were determined at 340 nm. The fit lines were obtained by linear regression with R^2^ greater than 0.988.


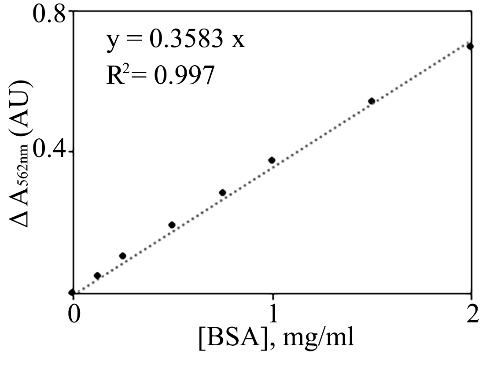


**Supplementary Figure 7. Bovine serum albumin standard curves for protein quantification by BCA.** BCA colour response curves produced using the Standard Test Tube Protocol (PierceTM BCA Protein Assay Kit) to quantify purified RrCβAA protein concentration. Raw data available at: <https://doi.org/10.6084/m9.figshare.12399611.v1>

**Supplementary Tables**

**Supplementary Table 1. Purification table of recombinant RrCβAA**

| **Purification step** | **Protein concentration (mg/mL)** | **Specific activity (U/mg)** | **Yield (%)** |
| --- | --- | --- | --- |
| Crude extract | 23.8 | 0.3 | 100.0 |
| His tag and concentration | 15.2 | 8.8 | 77.5 |
| Gel filtration and concentration | 12.6 | 13.4 | 65.2 |

**Supplementary Table 2. Temperature - activity relationship for RrCβAA**

The activity the recombinant RrCβAA enzyme was assessed between 25 and 70˚C. The reaction mixture was incubated at the mentioned temperature for 10 min. Afterwards, the reaction was started by the adding of enzyme and lasts for 15 min.

| **Temperature (°C)** | **Specific activity (U/mg)** | **Relative activity (%)** |
| --- | --- | --- |
| 25 | 2.7 ± 0.8 | 26±6 |
| 30 | 3.3±0.7 | 34±13 |
| 35 | 4.5±0.3 | 45±11 |
| 40 | 6.7±1.4 | 65±2 |
| 45 | 8.1±1.6 | 78±1 |
| 50 | 9.1±1.4 | 88±4 |
| 55 | 10.3±2.1 | 100±0 |
| 60 | 6.1±1.2 | 60±5 |
| 65 | 2.6±1.2 | 25±9 |
| 70 | 1.9±0.8 | 18±5 |

**Supplementary Table 3. Temperature - stability relationship for RrCβAA**

RrCβAA samples were incubated at various temperatures (25–70˚C) for 15 min in phosphate buffer, followed by incubation on ice. Residual activities were determined under the standard assay condition.

| **Temperature (°C)** | **Specific activity (U/mg)** | **Relative activity (%)** |
| --- | --- | --- |
| 25 | 11.7 ± 0.5 | 100 ± 0 |
| 30 | 11.8 ± 0.4 | 101 ± 5 |
| 35 | 11.1 ± 0.4 | 95 ± 6 |
| 40 | 11.2 ± 0.4 | 96 ± 7 |
| 45 | 9.3 ± 0.8 | 80 ± 4 |
| 50 | 8.4 ± 0.7 | 71 ± 6 |
| 55 | 5.5 ± 0.9 | 47 ± 7 |
| 60 | 5.3 ± 0.7 | 45 ± 4 |
| 65 | 2.4 ± 0.9 | 21 ± 8 |
| 70 | 0.8 ± 0.3 | 7 ± 2 |

**Supplementary Table 4. RrCβAA activity in the presence of cations and reducing agents.**

RrCβAA samples were incubated in the presence of 2 mM of each metal ion, DTT and EDTA, or 5 mM of DTNB and β-mercaptoethanol, for one hour at 4˚C. The activities of RrCβAA samples were determined under the standard assay condition. FeSO_4_ solution was prepared freshly for each experiment.

| **Sample** | **Specific activity (U/mg)** | **Relative activity (%)** |
| --- | --- | --- |
| As prepared | 13.6 ± 1.0 | 100 ± 7 |
| Na^+^ | 13.4 ± 0.7 | 99 ± 5 |
| K^+^ | 17.9 ± 2.0 | 131 ± 15 |
| Ca^2+^ | 18.3 ± 1.0 | 135 ± 8 |
| Mg^2+^ | 18.3 ± 1.3 | 135 ± 10 |
| Ba^2+^ | 13.0 ± 1.6 | 95 ± 12 |
| Sn^2+^ | 14.5 ± 1.0 | 107 ± 7 |
| Pb^2+^ | 13.3 ± 1.3 | 98 ± 10 |
| Fe^2+^ | 15.7 ± 2.2 | 116 ± 16 |
| Fe^3+^ | 14.1 ± 2.1 | 103 ± 15 |
| Mn^2+^ | 21.1 ± 2.5 | 155 ±18 |
| Ni^2+^ | 25.5 ± 3.1 | 188 ± 22 |
| Co^2+^ | 29.6 ± 4.0 | 218 ± 29 |
| Cd^2+^ | 28.6 ± 2.9 | 210 ± 21 |
| Zn^2+^ | 9.5 ± 0.6 | 70 ± 5 |
| Cu^2+^ | 6.7 ± 0.5 | 49 ± 4 |
| β-mercaptoethanol | 15.0 ± 1.0 | 110 ± 8 |
| DTT | 12.6 ± 0.8 | 93 ± 6 |
| DTNB | 4.2 ± 0.2 | 31 ± 2 |
| EDTA | 0.7 ± 0.3 | 5 ± 2 |

**Supplementary Table 5. Activity recovery of EDTA inactivated RrCβAA enzyme**

RrCβAA was incubated with 5 mM EDTA at 4˚C for one hour, followed by dialyses. Subsequently, samples of inactivated RrCβAA were incubated with the addition of 2 mM of each tested metal ion, followed by incubation at 4°C for one hour. The activity of the reactivated RrCβAA samples was determined under standard assay conditions.

| **Sample** | **Specific activity (U/mg)** | **Relative activity (%)** |
| --- | --- | --- |
| As prepared | 13.1 | 100 |
| EDTA inactivated | 0 ± 0 | 0 ± 0 |
| Na^+^ reactivated | 0.6 ± 0.1 | 4 ± 0 |
| K^+^ reactivated | 0.1 ± 0.1 | 1 ± 0 |
| Ca^2+^ reactivated | 1.1 ± 0.1 | 8 ± 1 |
| Mg^2+^ reactivated | 1.4 ± 0.1 | 11 ± 0 |
| Ba^2+^ reactivated | 0 ± 0 | 0 ± 0 |
| Sn^2+^ reactivated | 0 ± 0 | 0 ± 0 |
| Pb^2+^ reactivated | 0.3 ± 0.1 | 3 ± 0 |
| Fe^2+^ reactivated | 1.6 ± 0.1 | 13 ± 0 |
| Fe^3+^ reactivated | 1.9 ± 0.1 | 15 ± 1 |
| Mn^2+^ reactivated | 41.5 ± 4.5 | 319 ± 38 |
| Ni^2+^ reactivated | 42.4 ± 3.1 | 325 ± 24 |
| Co^2+^ reactivated | 54.7 ± 3.1 | 418 ± 24 |
| Cd^2+^ reactivated | 72.2 ± 4.6 | 552 ± 35 |
| Zn^2+^ reactivated | 0 ± 0 | 0 ± 0 |
| Cu^2+^ reactivated | 0 ± 0 | 0 ± 0 |

**Supplementary Table 6. Substrate preference of RrCβAA enzyme.**

A reaction mixture containing enzyme and different substrate (100 mM) was incubated at 40 ˚C for 10 min. The reaction was started by adding enzyme and was carried out for 15 min at 40 ˚C, pH 8.0.

| **Substrate** | **Specific activity (U/mg)** |
| --- | --- |
| N-carbamoyl-L-β-alanine | 11.4 ± 3.4 |
| N-carbamoyl-L-α-alanine | 6.1 ± 1.8 |
| N-carbamoyl-D-alanine | 0.0 ± 0.0 |
| N-carbamoyl-DL-alanine | 6.3 ± 1.1 |
| N-carbamoyl-L-valine | 0.0 ± 0.0 |
| N-carbamoyl-D-valine | 0.0 ± 0.0 |
| N-carbamoyl-DL-valine | 0.0 ± 0.0 |
| N-carbamoyl-L- β-phenyl-α-alanine | 0.2 ± 0.1 |
| N-carbamoyl-L-β-phenyl- β -alanine | 0.0 ± 0.0 |
| N-carbamoyl-D-β-phenyl-α-alanine | 0.0 ± 0.0 |
| N-carbamoyl-DL-β-phenyl-α-alanine | 0.3 ± 0.1 |
| N-carbamoyl-α-amino butyric acid | 5.5 ± 1.1 |
| N-carbamoyl-γ- amino butyric acid | 7.9 ± 1.0 |
| N-carbamoyl-L-leucine | 0.2 ± 0.1 |
| N-carbamoyl-L-methionine | 4.6 ± 1.1 |
| N-carbamoyl-glycine | 8.4 ± 2.1 |
| N-carbamoyl-3-(4-isopropoxyphenyl)-4-aminobutanoic acid | 0.0 ± 0.0 |
